# Ion-Sieving Dual-Network Hydrogel Electrolytes Couple Accelerated Ion Transport with Iodide Shuttle Suppression in Aqueous Zn–I_2_ Batteries

**DOI:** 10.1007/s40820-026-02216-6

**Published:** 2026-05-11

**Authors:** Ming Chen, Jia Cheng, Yixin Zhao, Wei Fu, Wen Li, Yunhai Zhu, Fanlu Meng

**Affiliations:** 1 School of Materials Science and Engineering, Ocean University of China, Qingdao, 266404 People’s Republic of China; 2 Key Laboratory of Marine Equipment Materials and Protection of Shandong Province, Qingdao, 266404 People’s Republic of China; 3 Tianfu Jiangxi Laboratory, Aerospace Special Power Source Technology Innovation Center, Chengdu, 641419 People’s Republic of China; 4 State Key Laboratory of New Textile Materials and Advanced Processing Technologies, Wuhan Textile University, Wuhan, 430200 People’s Republic of China

**Keywords:** Aqueous Zn–I_2_ batteries, Hydrogel electrolyte, Ion sieving, Polyiodide ion shuttle-free, Dendrite-free

## Abstract

Aqueous Zn–I_2_ batteries hold significant promise for large-scale energy storage applications; however, their practical implementation is constrained by coupled interfacial instabilities, including Zn dendrite growth, parasitic hydrogen evolution and polyiodide shuttling. Here we report a dual-network hydrogel electrolyte that stabilizes Zn and iodine chemistries through a synergistic, cross-scale design spanning molecular conformation regulation, charge microenvironment engineering and network topology control. By precisely tailoring the local charge microenvironment, the hydrogel electrostatically excludes polyiodide species to mitigate the shuttle effect, while simultaneously constructing low-energy pathways for Zn^2+^ migration, enabling differentiated ion regulation. The interconnected porous architecture further homogenizes ionic conduction and modulates three-dimensional Zn^2+^ flux at the Zn interface, delivering dendrite-free Zn plating/stripping for over 3500 and 800 h at areal capacities of 1 and 10 mAh cm^−2^, respectively. In Zn–I_2_ full cells, stable cycling is maintained for 20,000 cycles at a high rate of 10 C. This work establishes an integrated molecular-to-mesoscale electrolyte design strategy for advancing highly stable aqueous Zn–I_2_ batteries.

**Supplementary Information:**

The online version contains supplementary material available at 10.1007/s40820-026-02216-6.

## Highlights

- A dual-network hydrogel electrolyte integrates the mechanical robustness of polyacrylamide with functional groups from carboxymethyl chitosan (CMCS), overcoming the single-network trade-off between ionic conductivity and mechanical integrity.
- Introducing tert-butylamine suppresses amino group protonation and increases carboxyl group electronegativity, thereby directionally tuning the Zn^2+^ transport microenvironment.
- Uniform pores and high ionic conductivity promote a homogeneous Zn^2+^ flux to suppress dendrite growth, while electronegative carboxylate groups in CMCS inhibit polyiodide shuttling through electrostatic repulsion.

**Supplementary Information:**

The online version contains supplementary material available at 10.1007/s40820-026-02216-6.

## Graphical abstract

**Figure:**
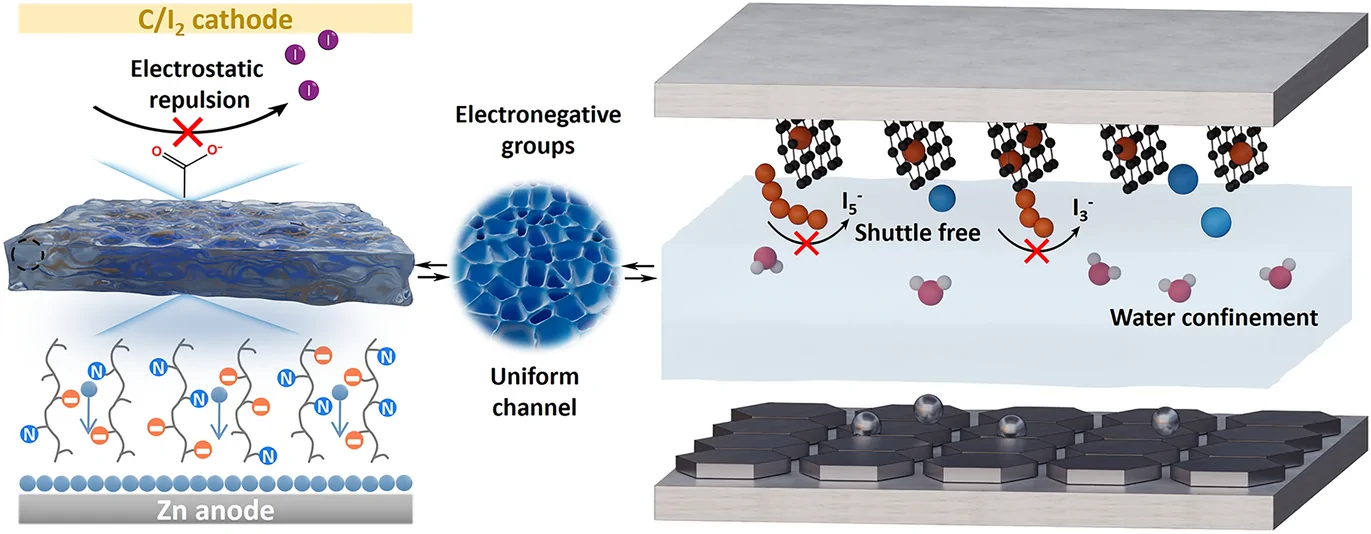

## Introduction

Aqueous Zn–I_2_ batteries have emerged as promising candidates for grid-scale energy storage, because they combine intrinsic safety, low materials cost and a relatively high, flat discharge plateau (~ 1.3 V vs. Zn/Zn^2+^) [1–4]. Nevertheless, their practical performance is dominated by coupled interfacial instabilities at both electrodes, which are strongly mediated by the electrolyte [5, 6]. At the Zn anode, spatially non-uniform Zn^2+^ transport and interfacial complex promote heterogeneous nucleation and growth, leading to dendrite formation and concurrent parasitic reactions such as hydrogen evolution and water-induced corrosion/passivation. In parallel, the iodine cathode undergoes multistep redox involving polyiodide intermediates (e.g., I_3_^−^, I_5_^−^) that readily dissolve and migrate; this shuttle process induces self-discharge and active material loss, while the migrated polyiodides severely corrode the Zn anode [7, 8]. A central challenge is that free water and the Zn^2+^ solvation environment concurrently governs Zn interfacial reactivity and polyiodide transport [9, 10]. High water activity sustains a strongly hydrated Zn^2+^ sheath and perturbs the interfacial electric field during desolvation, amplifying localized deposition and dendrite growth, while also facilitating water electrolysis and local pH fluctuations that accelerate side reactions [11–14]. Meanwhile, free water increases iodine solubility and lowers barriers for polyiodide diffusion, thereby intensifying shuttling and anode corrosion [15–17]. Quasi-solid-state hydrogel electrolytes offer a promising route by reducing water activity while maintaining ion conduction and enabling chemical/topological control over transport pathways [18]. However, an effective hydrogel for Zn–I_2_ batteries must accelerate Zn^2+^ migration (ideally lowering the desolvation barrier) while selectively suppressing polyiodide crossover, thereby overcoming the common conductivity–mechanical robustness trade-off and decoupling Zn^2+^ conduction from polyiodide shuttling [19–22].

Designing hydrogel electrolytes for Zn–I_2_ batteries requires balancing mechanical integrity, ion transport kinetics and interfacial selectivity within a single platform. The polymer skeleton must be sufficiently robust to maintain intimate contact and withstand repeated plating/stripping stress, yet it should also contain wide and continuous pathways to sustain fast ionic conduction at high current densities [21, 23–25]. This balance is difficult to achieve with single-network gels. For instance, highly crystalline and densely cross-linked poly(vinyl alcohol) networks that often restrict ion diffusion and are susceptible to microcrack formation under cyclic stress [26, 27]; polyacrylamide, lacking strong Zn^2+^-affinitive motifs, typically yields a low Zn^2+^ transference number, whereas amino-rich chitosan derivatives readily protonate in conventional electrolytes, creating cationic domains that repel Zn^2+^, raise the desolvation/migration barrier and intensify interfacial polarization. Such positively charged sites can also interact unfavorably with anionic polyiodides, compromising shuttle suppression and aggravating anode corrosion. Moreover, strengthening the gel by increasing cross-link density usually suppresses polymer segmental dynamics, narrows transport channels and penalizes ionic conductivity under high-rate operation [28, 29]. Collectively, these coupled constraints expose the intrinsic limitations of single-network architectures in simultaneously delivering robustness and selective, high-flux ion regulation [30], motivating dual-network hydrogel designs that decouple load bearing from transport and interfacial functions in Zn–I_2_ batteries [31].

**Fig. 1:**
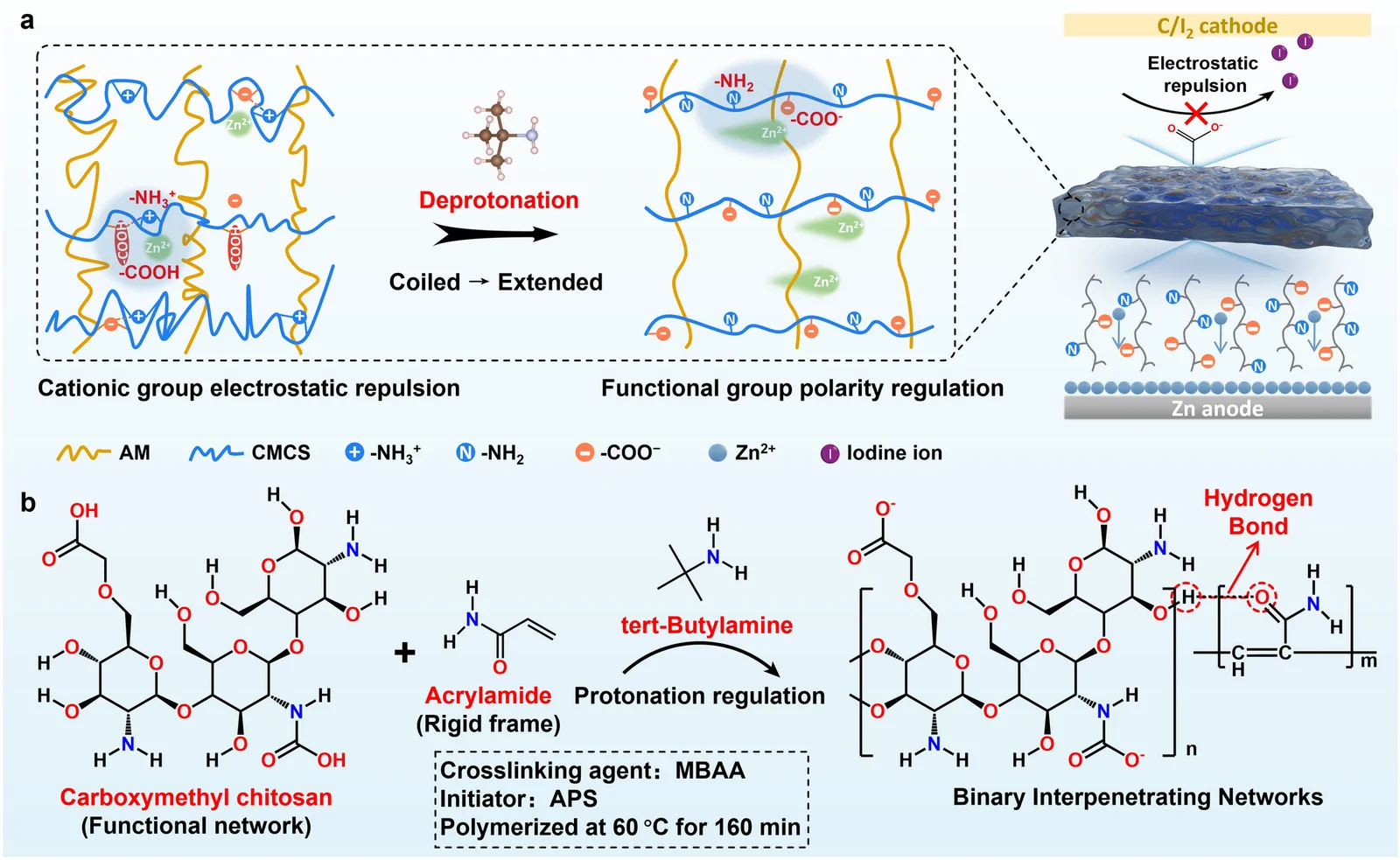
Synthesis strategy and fundamental functionalities of the PAM–CMCS–tB hydrogel electrolyte. **a** Dual-network structure synergistically regulates ion transport and stabilizes the electrode–electrolyte interface. **b** Hydrogen bonding between dual-network hydrogel and the directional regulation of tert-butylamine charge polarity

Herein, we engineer a dual-network hydrogel electrolyte that couples rapid Zn^2+^ transport with interfacial stabilization in aqueous Zn–I_2_ batteries (Fig. 1a). A three-dimensional polyacrylamide (PAM) scaffold provides mechanical resilience, whereas carboxymethyl chitosan (CMCS) introduces polar functionalities that form percolated conduction domains. Incorporating tert-butylamine (tB) tailors the local charge microenvironment by limiting amino group protonation and increasing the degree of carboxylate dissociation, thereby raising local negative charge density. This charge state tuning electrostatically excludes anionic polyiodide species to mitigate shuttling, while simultaneously creating a low-barrier pathway for Zn^2+^ motion. In addition, the mildly alkaline condition produced by tB hydrolysis promotes CMCS chain extension and exposes functional motifs, strengthening dynamic hydrogen-bonded cross-links with PAM and yielding a topologically uniform dual-network architecture hydrogel (denoted as PAM–CMCS–tB) that homogenizes three-dimensional Zn^2+^ flux and guides dendrite-free deposition (Fig. S1). Consequently, Zn symmetric cells employing the PAM–CMCS–tB hydrogel electrolyte operate stably for > 3500 h at 1 mAh cm^−2^ and for ~ 800 h at 10 mAh cm^−2^, whereas Zn–I_2_ full cells sustain 20,000 cycles at 10 C. Moreover, flexible devices maintain stable performance under bending, delivering 152.5 mAh g at 1 C. Collectively, this cross-scale electrolyte design paradigm integrating molecular conformation control, charge microenvironment engineering and network topology regulation for highly stable aqueous Zn–I_2_ batteries.

## Experimental Section

### Materials

Acrylamide (AM, C_3_H_5_NO, 99%), carboxymethyl chitosan (CMCS, degree of substitution ≥ 90%), ammonium persulfate (APS, H_8_N_2_O_8_S_2_, 98%), N,N′-methylenebis(acrylamide) (MBAA, C_7_H_10_N_2_O_2_, 99%), tert-butylamine (tB, C_4_H_11_N, 98%) and iodine (I_2_, 99.8%) are all purchased from Sigma-Aldrich. Zinc sulfate heptahydrate (ZnSO_4_·7H_2_O, 99%) and sodium sulfate (Na_2_SO_4_, 99%) are obtained from Sinopharm Chemical Reagent Co., Ltd. Zn foil (100 μm, 99.9%), Cu foil (100 μm, 99.9%) are purchased from Qingyuan Metal Co., Ltd. Stainless steel mesh (250 mesh) is purchased from Canrd Technology Co., Ltd. All chemicals and materials are used as received without further purification.

### Preparation of Several Hydrogel Electrolytes

For the preparation of PAM–CMCS–tB hydrogel electrolyte, 0.5 g CMCS and 2 g AM are dispersed in 10 mL of deionized water via ultrasonic treatment. Subsequently, 50 mg APS as an initiator and 20 mg MBAA as a cross-linking agent are added and stirred thoroughly to yield a transparent solution. Following this, 0.17 mL tB is introduced and vigorously mixed. The resulting solution is then poured into a PVDF mold and polymerized at 60 °C for 160 min under an argon atmosphere to form the hydrogel. Finally, the hydrogel is immersed in 2 M ZnSO_4_ solution to obtain the PAM–CMCS–tB hydrogel electrolyte. When tB is excluded while keeping all other synthesis parameters unchanged, PAM–CMCS hydrogel electrolyte is obtained using AM and CMCS as precursors, whereas PAM hydrogel electrolyte is obtained when only AM is used.

### Preparation of NC@I_2_ Cathodes

The NC material is prepared using a well-established protocol reported in previous studies [32]. Typically, 0.5 g iodine powder is weighed and thoroughly mixed with 0.5 g NC, which is synthesized according to previously reported methods. The mixture is sealed in a glass vial and heated at 90 °C for 6 h to obtain NC@I_2_ powder. Subsequently, the NC@I_2_ powder and Super P conductive carbon are combined in a mass ratio of 8:1. A small amount of PTFE (NC@I_2_ powder:PTFE is 8:1) emulsion is added, and the mixture is ground into a homogeneous slurry. The resulting slurry is uniformly coated onto a stainless steel mesh current collector and dried under vacuum at 40 °C for 12 h to form the NC@I_2_ cathode. The mass loading of iodine active material is maintained at approximately 1.5 mg cm^−2^.

### Assemble Pouch Cell

The NC@I_2_ cathode is prepared according to the method described in Sect. 2.3 and cut into a rectangular shape (3.5 cm × 4.5 cm), with an iodine active material loading of approximately 9.2 mg cm^−2^. The Zn foil is cut into a rectangular shape (3.7 cm × 4.7 cm), slightly larger than the cathode to ensure complete coverage. The PAM–CMCS–tB hydrogel is cut into a rectangular shape (4.0 cm × 5.0 cm), soaked in 2 M ZnSO_4_ solution for 2 h and then assembled sequentially with the Zn anode and NC@I_2_ cathode to form the gel electrolyte cell. After assembly, the pouch cell is sealed using aluminum–plastic film via thermal sealing.

### Material Characterization

Morphological characterization of the hydrogels and electrodes is conducted using a ZEISS Gemini SEM 300 scanning electron microscope. ^1^H NMR spectra are recorded using a Bruker DRX 400 NMR indicator. Fourier transform infrared (FTIR) spectra are acquired on a Thermo Scientific Nicolet iS50 spectrometer. X-ray photoelectron spectroscopy (XPS) measurements are taken on a Thermo Scientific K-Alpha instrument equipped with an Al K*α* excitation source (1486.6 eV). Pore volume is evaluated using an ASAP 2460 adsorption analyzer. Powder X-ray diffraction (XRD) patterns are recorded using a Bruker D8 Advance diffractometer. Small-angle X-ray scattering (SAXS) data are collected using an Anton paar Saxsess MC2 system. The surface roughness of the Zn anodes after cycling is evaluated using a KEYENCE VK-X250 3D laser confocal microscope. Zeta potential measurements are taken using a Malvern Zetasizer Nano ZS (ZEN3600). Ultraviolet–visible (UV–Vis) absorption spectra are recorded on a Shimadzu UV-3600 spectrophotometer. In situ confocal Raman spectra are obtained using a Renishaw inVia Raman microscope.

### Electrochemical Measurements

Electrochemical performance is evaluated using CR2032-type coin cells assembled with various hydrogel electrolytes as the electrolyte component. Symmetric cells are constructed using zinc foil as both the working and counter electrodes, while Zn–Cu half-cells are fabricated with zinc and copper foils as the respective electrodes. Full cells are assembled with zinc foil as the anode and NC@I_2_ as the cathode. Battery testing is conducted using a LAND CT2001A battery testing system (Wuhan LANHE). Electrochemical measurements including linear sweep voltammetry (LSV), Tafel plots, chronoamperometry (CA), electrochemical impedance spectroscopy (EIS) and cyclic voltammetry (CV) are taken using a CHI760E electrochemical workstation (Shanghai Chenhua). For LSV tests, using zinc foil as the working electrode and a stainless steel plate as both the counter and reference electrodes. The scan rate is set to 1 mV s^−1^. Tafel measurements are taken at a scan rate of 5 mV s^−1^, while CA measurements are taken at a constant potential of 150 mV.

### Computational Details

#### Molecular Dynamics Simulation

Molecular dynamics (MD) simulations are conducted using Materials Studio, with the COMPASS II force field applied to all simulated systems. The velocity Verlet algorithm is employed to integrate the equations of motion for all atoms. Initial atomic velocities are randomly assigned, and a time step of 1 fs is used. A cutoff distance of 12.5 Å is applied for non-bonded van der Waals interactions. Electrostatic interactions are computed using the Ewald summation method. The chitosan and polyacrylamide molecular chains consisted of 8 and 16 monomer units, respectively. Following model construction, an annealing process is applied to relax polymer entanglement, prevent excessive chain bending and improve structural stability. The annealing protocol comprised five cycles, each involving heating from 300 to 500 K followed by cooling back to 300 K. Subsequently, NPT ensemble simulations are carried out at 298 K and 1 atm for 2 ns using the final equilibrated configurations.

#### Density Functional Theory Calculations

All calculations are performed based on density functional theory (DFT) using the Vienna ab initio Simulation Package (VASP) [33, 34]. The exchange–correlation interactions are described using the Perdew–Burke–Ernzerhof (PBE) functional within the generalized gradient approximation (GGA), and the interactions between core and valence electrons are treated using the projector augmented-wave (PAW) method [35–37]. A plane-wave energy cutoff of 480 eV is employed, with Brillouin zone sampling performed using a 1 × 3 × 3 Monkhorst–Pack k-point grid. To eliminate spurious interactions caused by periodic boundary conditions, a vacuum layer of 15 Å is introduced normal to the surface plane. Structural relaxation is carried out until the total energy change converged to 1.0 × 10^−4^ eV, and the residual atomic forces are less than 0.02 eV Å^−1^. The adsorption energy (*E*_ad_) is calculated using the following equation:

$${E}_{\mathrm{a}\mathrm{d}}={E}_{\mathrm{a}\mathrm{b}}-{E}_{a}-{E}_{b}$$

where *E*_ab_ represents the total energy of the adsorption system containing both the adsorbate and the substrate, *E*_*a*_ denotes the total energy of the isolated adsorbate and *E*_*b*_ corresponds to the total energy of the clean substrate.

The molecular electrostatic potential is calculated using the Multiwfn program and visualized with VMD [38–40].

## Results and Discussion

### Dual-Network Hydrogel Design and Structural Characterization

**Fig. 2:**
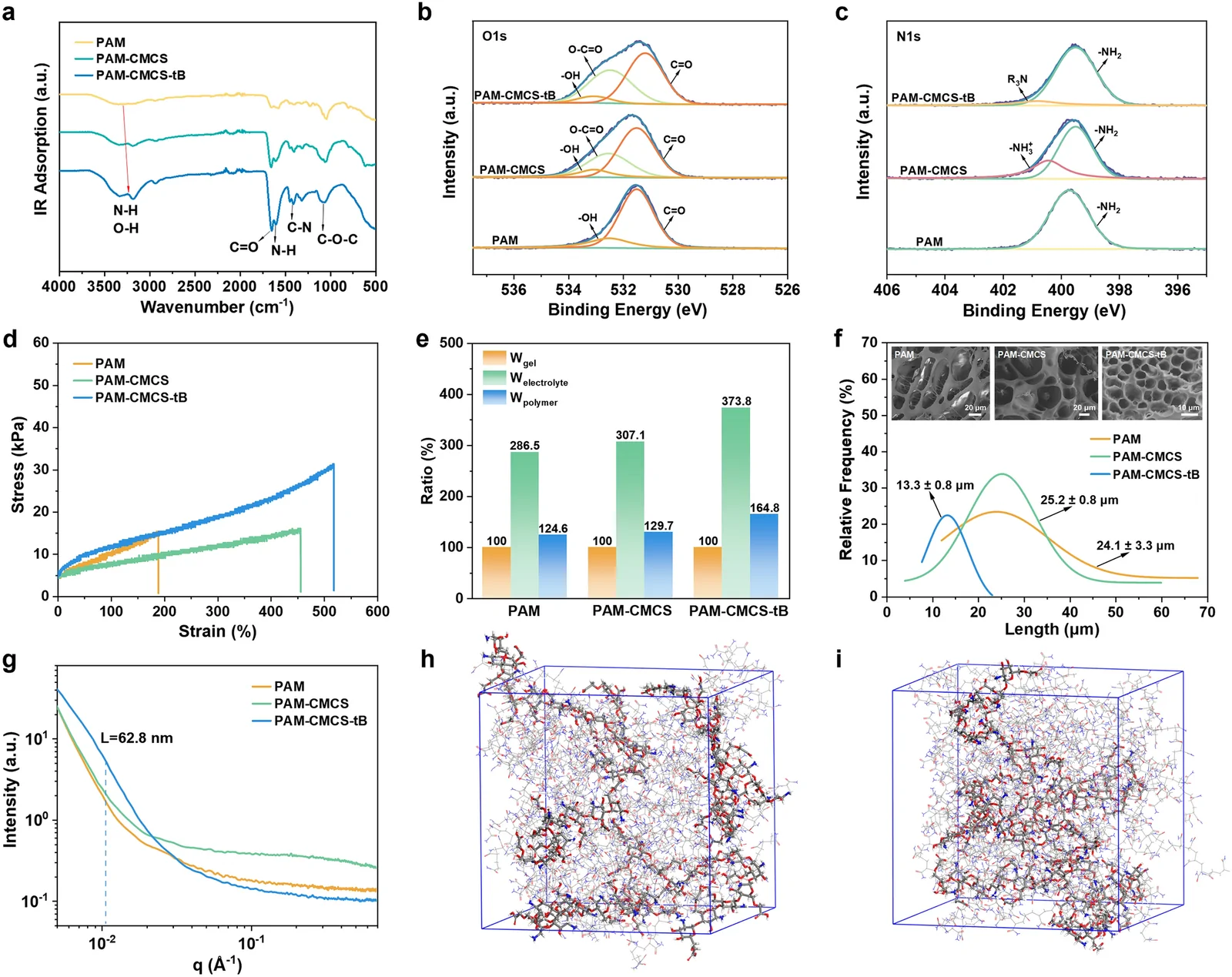
Structural and physicochemical characterization of different hydrogel electrolytes. **a** FTIR spectra. **b, c** XPS spectra showing O 1*s* and N 1*s* peaks. **d** Stress–strain curves. **e** Composition changes of different hydrogel electrolytes in 2 M ZnSO_4_. **f** Pore size distribution analyzed from SEM images of the hydrogel electrolytes. **g** Small-angle X-ray scattering (SAXS) spectrum. **h, i** Molecular dynamics simulations showing the structural configuration of the PAM–CMCS–tB and PAM–CMCS hydrogel network

The dual-network hydrogel, prepared as shown in Fig. 1b, comprises acrylamide as a rigid backbone that provides sufficient mechanical strength and carboxymethyl chitosan as the functional network, with polymer chains interconnected via hydrogen bonds. The carboxyl content of the hydrogel is quantitatively determined by NMR and found to be approximately 0.23 mmol mL^−1^ (Fig. S2). Consequently, this hydrogel balances water retention and functional performance, enabling ion migration in liquid phases and facilitating rapid Zn^2+^ transport along polymer chain segments. The bonding mechanism within the hydrogel structure is investigated using IR spectroscopy (Fig. 2a). Characteristic peaks corresponding to C–O–C stretching vibrations, attributable to PAM and CMCS, appear at 1074 cm^−1^, accompanied by enhanced *v*(C–N), *β*(N–H) and *v*(C=O) signals at 1412, 1605 and 1648 cm^−1^, respectively, after introducing CMCS. In addition, the broad *v*(N–H) and *v*(O–H) bands at approximately 3184 and 3338 cm^−1^ exhibit a red shift, indicating the formation of hydrogen-bonded cross-links between PAM and CMCS [41]. XPS spectra further corroborate the compositional and chemical state evolution (Figs. 2b, c and S3). The O–C=O component associated with carboxyl groups is markedly enhanced after incorporating CMCS. Moreover, deprotonation by tB increases the number of exposed _–_COO^−^ groups, leading to a further rise in the O–C=O signal intensity. Similar changes are observed in the N 1*s* spectrum, except for a persistent -R_3_N signal at 400.82 eV. Notably, –NH_2_ becomes the dominant nitrogen species in PAM–CMCS–tB, replacing the mixed presence of –NH_2_/–NH_3_^+^ distribution detected in PAM–CMCS [42, 43].

The tensile performance of PAM–CMCS–tB (Fig. 2d) reveals that, compared with the simple covalent cross-linking PAM network, the dual-network hydrogel exhibits enhanced fracture toughness (Table S1). Moreover, the highly stretchable hydrogel can continuously accommodate electrode deformation during cycling, thereby maintaining continuous ion transport pathways and preventing performance degradation caused by structural fatigue of the hydrogel (Fig. S4). The swelling behavior of the hydrogel electrolyte is evaluated in water and 2 M ZnSO_4_ solutions (Figs. 2e and S5), with PAM–CMCS–tB exhibiting the highest solvent uptake. Notably, stabilizing the salts within the hydrogel network helps sustain a homogeneous ion transport pathway [44]. Subsequently, aging experiments conducted in air (Fig. S6) reveal that PAM–CMCS–tB hydrogel retains the most water after the initial evaporation of free water over 72 h of resting, which is attributed to the anchoring effect of hydrophilic groups within the polymer chain network [45]. We performed a pore size statistical analysis based on SEM images of PAM, PAM–CMCS and PAM–CMCS–tB gels, with at least 50 pores randomly measured for each sample. The maximum pore diameter was measured using ImageJ software, followed by statistical analysis of the collected data. Fig. 2f shows that PAM–CMCS–tB exhibits an average pore size of ~ 13.3 μm. Under deprotonation regulated by tB, the CMCS chains undergo conformational stretching, thereby exposing additional multifunctional groups and forming a more homogeneous network cross-linking structure (Figs. S7 and S8). This homogeneous pore structure generates continuous ion transport channels under topological constraints, thereby reducing Zn^2+^ diffusion resistance and balances its migration rate to mitigate concentration gradients at the electrode–electrolyte interface [46]. Small-angle X-ray scattering (SAXS) is employed to probe the molecular network distribution within the hydrogel (Fig. 2g). The scattering profile of the PAM–CMCS–tB hydrogel exhibits distinct peaks in the low-angle region, indicating a microscopically periodic structural arrangement (Fig. S9) [47]. Using Guinier analysis, we calculated the radius of gyration (Rg), which reflects the average size of the polymer network structure. The Rg value of PAM–CMCS–tB (35.6 nm) is smaller than that of PAM (45.4 nm) and PAM–CMCS (43.9 nm), and the distribution is narrow, indicating a more compact and uniform gel network structure (Fig. S10). In addition, the influence of tB on CMCS conformational behavior is examined via molecular dynamics (MD) simulations (Figs. 2h, i and S11). After 2 ns, tB-induced deprotonation enables the CMCS segments within the PAM–CMCS–tB hydrogel to maintain an extended conformation. By contrast, in the absence of tB (PAM–CMCS hydrogel), the CMCS segments undergo pronounced contraction due to strong electrostatic interactions between –NH_3_^+^ and –COO^−^ groups. Furthermore, the radius of gyration of CMCS chains decreases (Fig. S12), indicating that the PAM–CMCS hydrogel cannot preserve an extended and uniformly distributed architecture.

### Regulation of Zn^2+^ Transport and Dendrite-Free Deposition

**Fig. 3:**
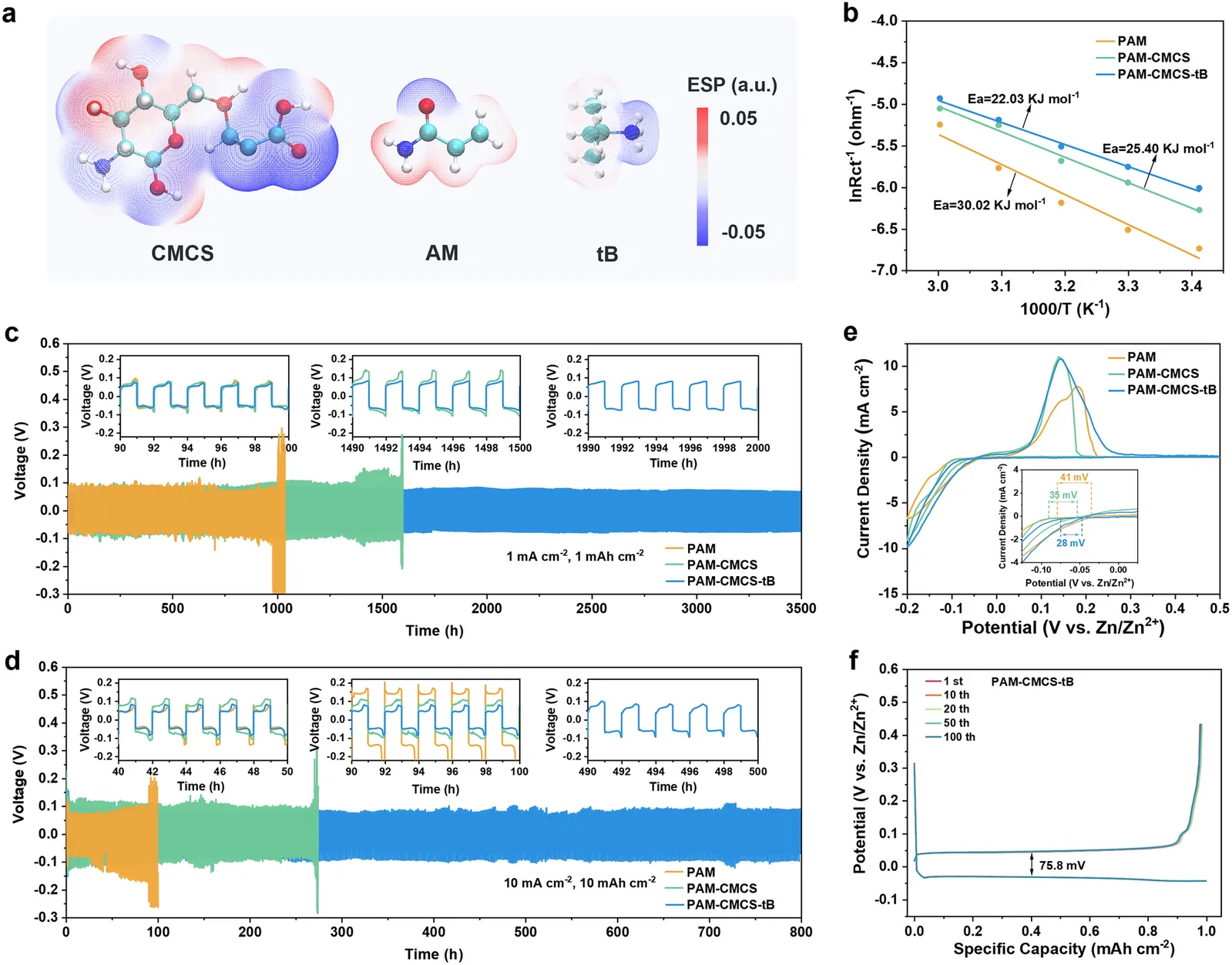
Regulation mechanism of PAM–CMCS–tB hydrogel electrolyte on Zn electrochemical behavior. **a** Electrostatic potential distribution of polymer chain components. **b** Arrhenius curves comparing activation energy (Ea). **c, d** Cycling performance of Zn//Zn symmetric cells at current densities of 1 mA cm^−2^ and 10 mA cm^−2^, respectively, with matched areal capacities of 1 mAh cm^−2^ and 10 mAh cm^−2^. **e** CV curves of Zn//Cu half-cells. **f** Zn deposition/stripping voltage profiles in half-cell tests

To evaluate the practical electrochemical performance of the hydrogel electrolytes, Zn//Zn symmetric cells are assembled using different gel formulations, and their stability windows are assessed by linear sweep voltammetry (Fig. S13). Owing to the abundance of unbound free water molecules in the PAM network, these molecules directly participate in interfacial reactions, giving rise to pronounced HER and OER activities. When CMCS is incorporated to form a dual-network hydrogel, extensive hydrogen bonds convert free water into bound water; subsequent introduction of tB further enhances water binding within the more homogeneous polymer chain network [48]. This trend is supported by the Tafel analysis (Fig. S14), where the corrosion current density of the Zn anode in the PAM–CMCS–tB hydrogel electrolyte is 0.41 mA cm^−2^, markedly lower than the 1.21 mA cm^−2^ observed in the PAM hydrogel, indicating effectively suppressed corrosion kinetics and mitigated parasitic reactions [49]. Additionally, the potential ion transport driving sites within the dual-network matrix are inferred using electrostatic potential mapping (Fig. 3a). A high density of negative charges is localized on the –COO^−^ groups, generating a Donnan potential that influences the Zn^2+^ distribution and migration through the porous polymer framework [50]. In addition, the electrostatic potential map of the tB molecule clearly shows that the lone pair region on the nitrogen atom exhibits a strong negative potential. This pronounced negative potential underlies its high basicity and provides the driving force for subsequent regulation of the CMCS charge state. Consistently, the zeta potential in the PAM–CMCS–tB system is the most negative (−4.47 mV; Fig. S15), suggesting that the mildly alkaline environment associated with tB effectively increases the electronegativity of the –COO^−^ groups. The negatively charged, electron-rich groups strengthen field-driven ion transport, increasing ionic conductivity from 7.2 mS cm^−1^ for the PAM hydrogel to 18.1 mS cm^−1^ for PAM–CMCS–tB (Fig. S16) [51]. Furthermore, conformational stretching of polymer chain segments exposes additional functional groups, which facilitates Zn^2+^ hopping/coordination exchange across the network and lowers the apparent activation energy (Ea) for ion transport (Figs. 3b and S17).

The cycling stability of the hydrogel electrolyte is evaluated by assembling Zn//Zn symmetric cells. Under a deposition condition of 1 mA cm^−2^/1 mAh cm^−2^, the cell employing the PAM–CMCS–tB hydrogel electrolyte delivers a cycle life exceeding 3500 h. In contrast, the cells assembled with PAM and PAM–CMCS hydrogel electrolytes fail after approximately 970 and 1500 h (Fig. 3c), respectively. This comparison demonstrates that the PAM–CMCS–tB hydrogel electrolyte stabilizes electrode–electrolyte interface reactions by homogenizing Zn^2+^ transport and significantly improving the uniformity of Zn deposition [52]. Even at higher 10 mA cm^−2^/10 mAh cm^−2^, the PAM–CMCS–tB hydrogel can provide sufficient Zn^2+^ supply to sustain rapid depletion at the electrode surface, ensuring cycling with controlled voltage hysteresis for over 800 h (Fig. 3d). The PAM–CMCS–tB hydrogel electrolyte also enables stable Zn plating/stripping across a broad current density range from 0.1 to 10 mA cm^−2^ (Fig. S18).

To probe Zn nucleation and growth, Zn//Cu half-cells are assembled and cyclic voltammetry (CV) profiles are analyzed (Fig. 3e). A smaller nucleation overpotential (28 mV) indicates that the PAM–CMCS–tB promotes a higher nucleation density and faster nucleation kinetics, leading to a finer and more homogeneous deposit and thus a reduced propensity for dendrite growth. Repeated Zn plating/stripping experiments are conducted at a fixed areal capacity of 1 mAh cm^−2^, with the results shown in Figs. 3f and S19. The overpotential in PAM–CMCS–tB hydrogel electrolyte (75.8 mV) is lower than that in PAM (91 mV) and PAM–CMCS (84.2 mV). After 500 cycles, the half-cell with PAM–CMCS–tB maintains a Coulombic efficiency (CE) of 99.5% (Fig. S20). These results from symmetric cell and half-cell tests demonstrate that the PAM–CMCS–tB hydrogel electrolyte significantly enhances the interfacial stability of the Zn anode.

**Fig. 4:**
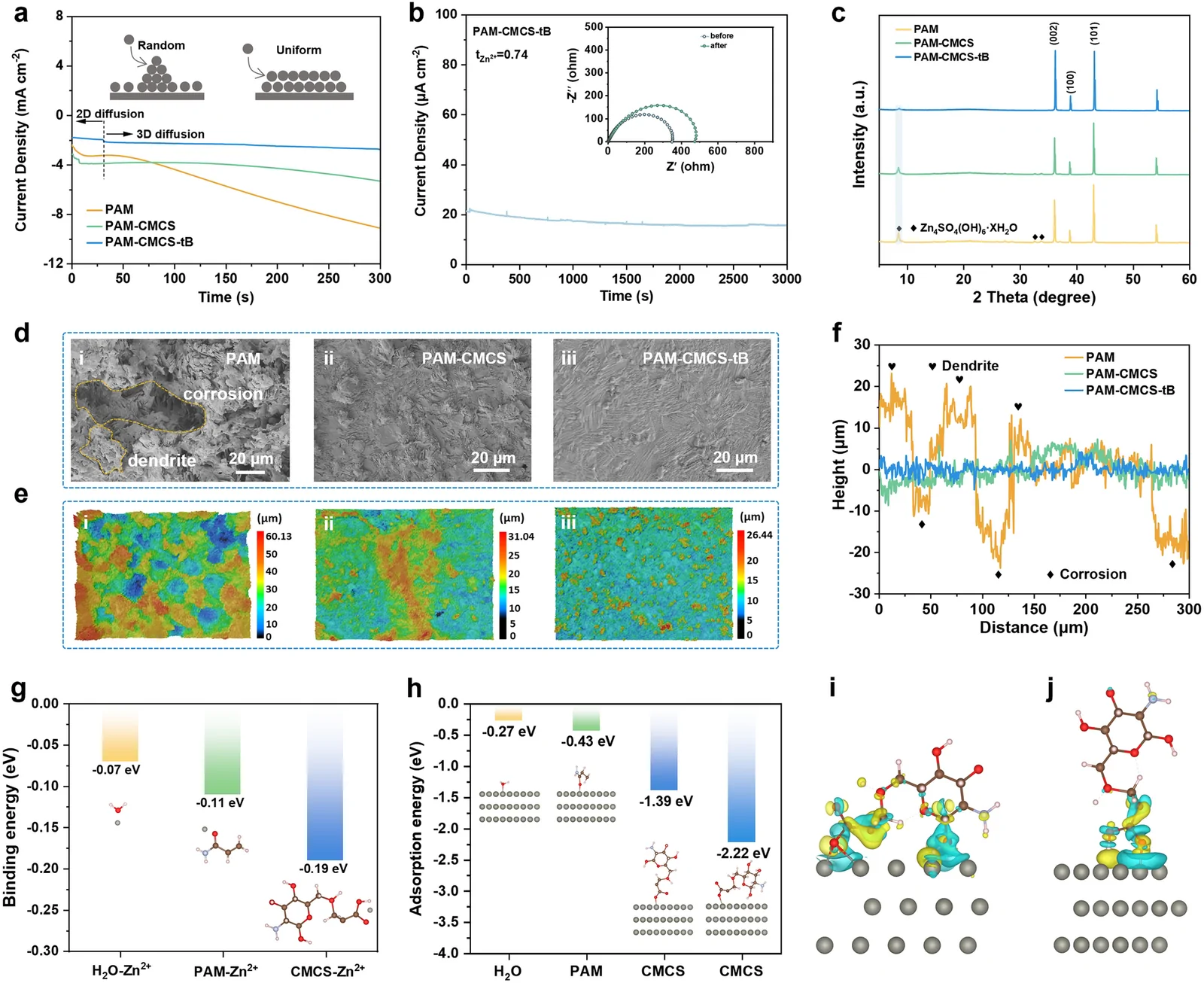
Effect of PAM–CMCS–tB hydrogel electrolyte on the nucleation and growth behavior of Zn anode. **a** Chronoamperometric curves. **b** Chronoamperometric curve recorded at a constant potential of 20 mV, with the inset showing Nyquist plots obtained before and after polarization. **c** XRD patterns of Zn anodes after cycling. **d** SEM images of Zn anode surfaces after 100 cycles of plating/stripping at 1 mAh cm^−2^ for PAM, PAM–CMCS and PAM–CMCS–tB hydrogel electrolytes, respectively. **e** Corresponding CLSM 3D surface morphology images. **f** Surface roughness profiles of Zn anodes after cycling. **g** Calculated binding energies between Zn^2+^ and various functional groups in the polymer chain. **h** Simulated adsorption configurations and energies of Zn^2+^ on the Zn (002) crystal plane. The charge density difference of CMCS adsorbed on Zn substrate in **i** transverse and **j** longitudinal directions

To clarify the impact of PAM–CMCS–tB on Zn deposition and interfacial evolution, chronoamperometric (CA) testing is employed first. As shown in Fig. 4a, the current density on the electrode with the PAM–CMCS–tB hydrogel remains nearly constant, indicating that Zn^2+^ nucleation and growth are governed by a homogeneous and stable three-dimensional diffusion process. In contrast, electrodes using PAM and PAM–CMCS hydrogels show a continuously increasing current density during the test, accompanied by a reduced nucleation density at high current densities. Under these conditions, Zn^2+^ growth preferentially proceeds through localized two-dimensional diffusion and finally forming dendritic or mossy structure. In addition to facilitating Zn^2+^ translocation through a uniformly distributed porous architecture, the fully exposed electronegative coordination sites along the polymer chains enhance Zn^2+^ trapping, increasing the Zn^2+^ transference number from 0.47 and 0.62 for PAM and PAM–CMCS, respectively, to 0.74 for PAM–CMCS–tB (Figs. 4b and S21). The higher proportion of Zn^2+^ charge carriers further mitigates concentration polarization and thereby improves rate capability.

XRD patterns (Fig. 4c) demonstrate that the electrode cycled with the PAM–CMCS–tB hydrogel exhibits the highest I_(002)_/I_(100)_ intensity ratio, and no appreciable formation of zinc hydroxide sulfate by-products is detected. The surface morphology of the Zn anode after cycling is examined using SEM combined with confocal laser scanning microscopy (CLSM). The Zn surface with the PAM hydrogel display numerous irregular deposits and corrosion pits (Figs. 4d and S22), whereas in the dual-network hydrogel system the surface gradually evolves into a smoother and denser deposition layer. This improvement can be attributed to the reduction of active water in the gel, which effectively mitigates side-reaction-induced corrosion. We conducted a systematic statistical analysis of the surface roughness of Zn electrodes after cycling. For each electrode, five different regions (100 μm × 100 μm) were selected for confocal laser scanning microscopy (CLSM) measurements. The average roughness (*R*_a_) and maximum height (*R*_z_) were calculated for each region (Fig. S23). Three-dimensional surface images further confirm the enhanced uniformity of Zn deposition and stripping processes (Figs. 4e and S24), and the decreased roughness correspondingly lowers the risk of short-circuiting, thereby prolonging cycle life (Fig. 4f).

To probe the interaction between polymer components and Zn^2+^, density functional theory (DFT) calculations are performed. The binding energy between –COO^−^ groups in CMCS and Zn^2+^ (−0.19 eV) is higher than that of amide groups on AM (−0.11 eV), indicating that Zn^2+^ primarily coordinated with –COO^−^ groups in the hydrogel network, which supports rapid capture and translocation (Fig. 4g) [53]. Furthermore, the binding energy between the polymer functional groups and Zn^2+^ exceeds those between water molecules and Zn^2+^, which facilitates the desolvation of hydrated zinc ions. According to the preferential adsorption model, the adsorption energy of CMCS molecules on the zinc surface in various configurations is consistently higher than those of water molecules (Figs. 4h and S25), indicating that the hydrogel electrolyte establishes a water-deficient interfacial environment that suppresses hydrogen evolution-induced corrosion. Notably, CMCS exhibits strong interactions with the Zn(002) crystal plane, as reflected by its adsorption energies in the parallel (−2.22 eV) and vertical (−1.39 eV) orientations. Differential charge density analyses further demonstrate that the carboxyl groups serve as electron transfer bridges, enabling charge redistribution between CMCS molecules and the electrode interface (Figs. 4i, j) [50].

### Suppression of Polyiodide Shuttling

**Fig. 5:**
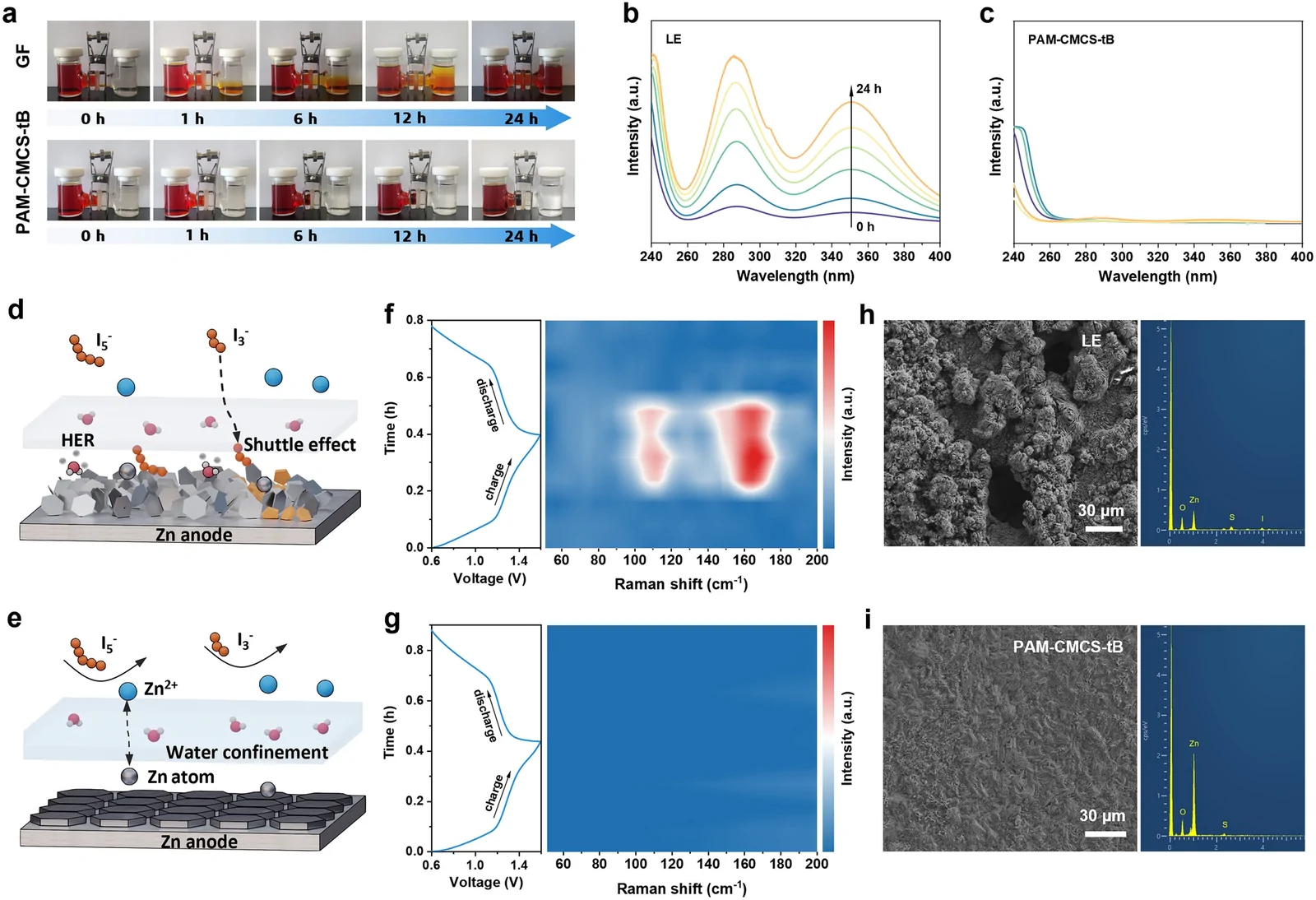
Inhibition of polyiodide ion shuttling by the PAM–CMCS–tB hydrogel electrolyte. **a** Optical images of the polyiodide ion diffusion in H-type cells separated by either a glass fiber membrane or PAM–CMCS–tB hydrogel. UV–Vis absorption spectra of the solution in the right chamber at different time intervals using **b** a glass fiber membrane and **c** PAM–CMCS–tB hydrogel. Effects of different electrolytes on Zn–I_2_ full battery cycling: **d** liquid electrolyte and **e** hydrogel electrolyte. Voltage–time profiles and corresponding in situ Raman spectra of Zn–I_2_ batteries during one charge–discharge cycle using **f** a liquid electrolyte and **g** the PAM–CMCS–tB hydrogel electrolyte. SEM images of Zn anode after cycling with **h** the liquid electrolyte and **i** the PAM–CMCS–tB hydrogel electrolyte

The optimization of the hydrogel for the iodine-based cathode is also investigated. An H-type electrolytic cell, containing a polyiodide ion solution and deionized water in separate chambers, is used to evaluate the polyiodide-blocking ability of the PAM–CMCS–tB hydrogel in comparison with the glass fiber separator and PAM–CMCS hydrogel (Figs. 5a and S26). With the glass fiber separator, discernible polyiodide diffusion occurred within just 1h and intensifies over the following 24 h. When the PAM–CMCS hydrogel is used, the right chamber also exhibited noticeable polyiodide diffusion over time. By contrast, when the PAM–CMCS–tB hydrogel serves as the separator, no obvious color change is observed even after 24 h. To quantify this behavior, UV–Vis absorption spectra of the solution in the right chamber are analyzed at different time intervals, as shown in Figs. 5b, c and S27, S28. With the glass fiber separator or PAM–CMCS hydrogel, the intensities of the I_3_^−^ and I_5_^−^ absorption peaks gradually increase over time. In contrast, no detectable I_3_^−^ or I_5_^−^ peaks appear when the PAM–CMCS–tB hydrogel is used, demonstrating effective suppression of polyiodide shuttling [54]. The underlying mechanism is further probed by theoretical calculations (Fig. S29). Simulations of the interaction between I_3_^−^ and I_5_^−^ and the –COO^−^ groups in CMCS indicate that substantial energy barriers must be overcome for polyiodide to approach the hydrogel matrix; moreover, the barrier increases as the distance decreases. This trend suggests that polyiodide transport through the polymer network toward the Zn anode is thermodynamically unfavorable [55, 56].

In situ Raman spectroscopy is then employed to verify the function of the hydrogel in an operating cell (Figs. 5f, g and S30, S31). During a charge–discharge cycle, as charging progressed, I_3_^−^-related peaks at approximately 110 cm^−1^ and I_5_^−^-related peaks at 165 cm^−1^ is detected on the surface of the Zn anode when using a glass fiber separator combined with a liquid electrolyte, consistent with polyiodide crossover that can accelerate Zn corrosion [57]. In contrast, benefiting from the immobilization of polyiodide ions by the PAM–CMCS–tB hydrogel, no I_3_^−^ or I_5_^−^ species are detected on the surface of the Zn anode throughout the charge–discharge cycle. Scanning electron microscopy (SEM) images reveal that Zn cycled with the glass fiber separator and liquid electrolyte suffers from corrosion and uneven deposition (Fig. 5h), whereas Zn cycled with the PAM–CMCS–tB hydrogel maintains a uniform and dense morphology (Fig. 5i).

### Electrochemical Performance of Zn–I_2_ Full Cells

**Fig. 6:**
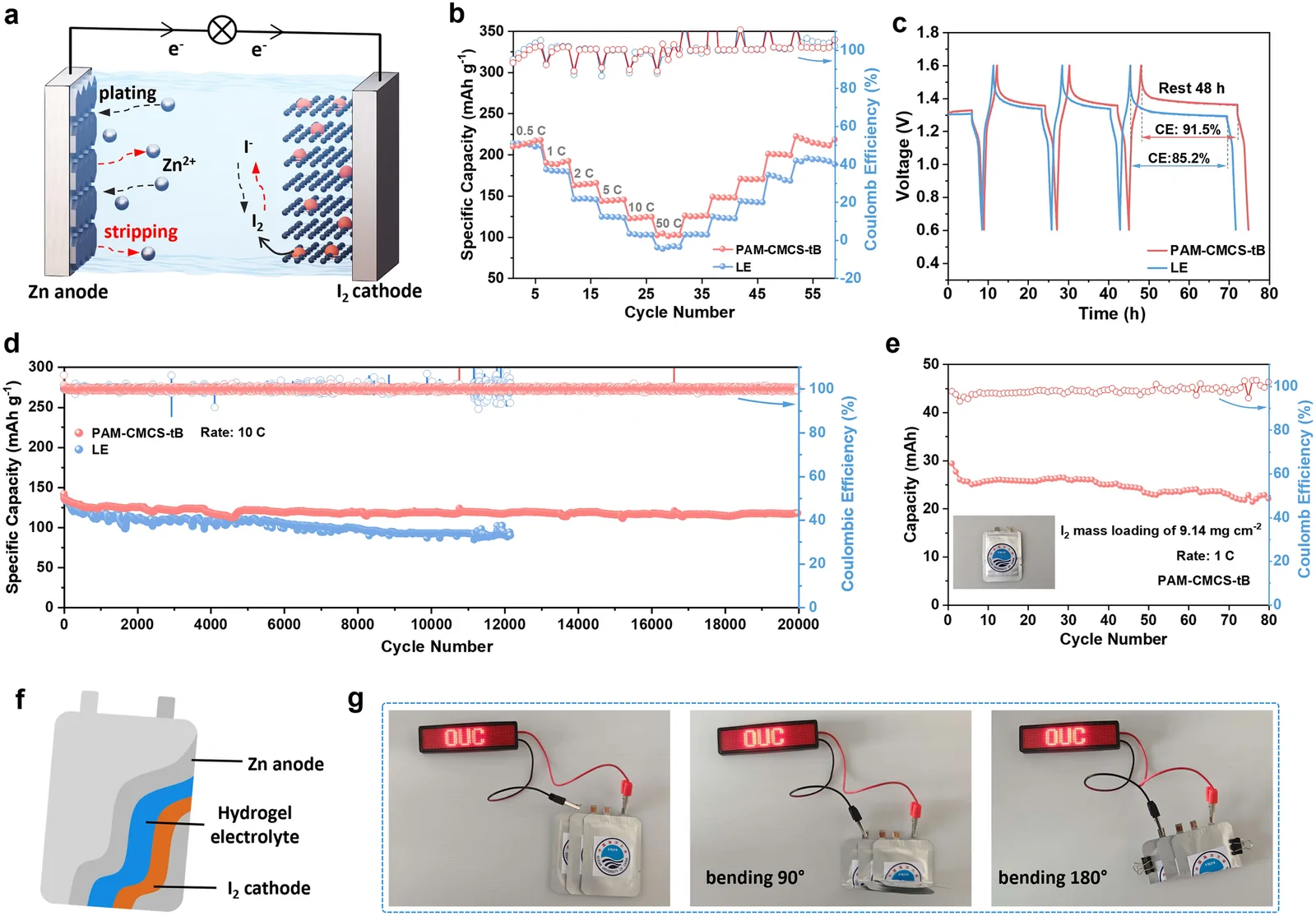
Electrochemical performance of Zn–I_2_ full cells. **a** Schematic of aqueous Zn–I_2_ cells. **b** Rate capability. **c** Capacity retention under different rest intervals. **d** Cycling performance at a high rate of 10 C. **e** Cycling stability of the pouch cell at 1 C rate. **f** Schematic of Zn–I_2_ pouch cells assembly. **g** Optical images of the LED device powered by a PAM–CMCS–tB hydrogel-based pouch cell

Zn–I_2_ full cells are assembled to evaluate the practical electrochemical performance of the PAM–CMCS–tB hydrogel, using iodine-loaded activated carbon as the cathode (Figs. 6a and S32). The cyclic voltammetry (CV) curves record at different scan rates are shown in Fig. S33, corresponding to a pair of reversible redox peaks associate with the two-electron conversion of iodine species. Compared with the liquid electrolyte, the full cell employing the PAM–CMCS–tB hydrogel electrolyte exhibits a higher specific capacity at high current densities (Fig. 6b). Moreover, after cycling at various current rates, the capacity recovers to 234.4 mAh g^−1^ when the current rate is returned to 0.5 C, demonstrating excellent rate capability and cycling reversibility (Fig. S34). According to the rate performance data shown in Fig. 6b, the specific discharge capacity of the PAM–CMCS–tB gel electrolyte at 0.5 C is 234 mAh g^−1^, and the average discharge voltage of the Zn–I_2_ battery is approximately 1.25 V. Based on these values, the gravimetric energy density calculated with respect to the iodine active material is 292.5 Wh kg^−1^. Electrochemical impedance spectroscopy (EIS) results further reveal the charge transfer resistance (*R*_ct_) of only 96.7 Ω (Fig. S35) for the cell with PAM–CMCS–tB, suggesting that the hydrogel maintains good interfacial contact, promoted efficient Zn^2+^ migration and effectively reduced interfacial impedance. In addition, the effective suppression of polyiodide shuttle effect mitigated the self-discharge issue (Fig. 6c). After several intermittent resting periods, the cell with PAM–CMCS–tB retains 91.5% of its initial capacity, outperforming the liquid electrolyte cell (85.2%). The continuous detection results of open-circuit voltage changes showed that the OCV of the liquid electrolyte with glass fiber separator decreased from 1.31 to 1.28 V, corresponding to a decay rate of 2.5 mV h^−1^. In contrast, the OCV of the PAM–CMCS–tB system decreased from 1.32 to 1.31 V, with a significantly lower decay rate of 0.83 mV h^−1^, representing a 66.8% reduction (Fig. S36). At 1 C, the PAM–CMCS–tB full cell delivers an initial specific capacity of 178.7 mAh g^−1^ and retained 87.2% of its maximum capacity (187.2 mAh g^−1^) after 2000 cycles (Figs. S37 and S38). In contrast, cells using liquid electrolytes exhibit rapid capacity fading after ~ 1550 cycles due to pronounced side reactions. Notably, the PAM–CMCS–tB-based cell sustains > 20,000 cycles at a high rate of 10 C, ultimately maintaining a specific capacity of 117.6 mAh g^−1^ with 89.1% capacity retention, whereas the liquid electrolyte counterpart shows continuous deterioration throughout the cycling process (Figs. 6d and S39). As shown in Fig. 6e, the assembled Zn/PAM–CMCS–tB/I_2_ pouch cell delivers a high specific capacity of 203.4 mAh g at 1 C and retains 152.5 mAh g after 80 cycles (Figs. S40 and S41). The pouch device can stably power an LED device even under a 180° bending condition (Fig. 6g). Following repeated bending and electrochemical cycling, the pouch cell continues to operate an environmental monitoring unit (Fig. S42), underscoring the potential of the PAM–CMCS–tB hydrogel electrolyte for flexible and wearable electronics. Compared with previously reported Zn–I_2_ batteries (Table S2), those employing the PAM–CMCS–tB hydrogel electrolyte exhibit superior overall electrochemical performance.

## Conclusion

In summary, we report a dual-network hydrogel electrolyte that delivers concurrent acceleration of Zn^2+^ transport and stabilization of interfacial reactions in aqueous Zn–I_2_ batteries through a cross-scale strategy spanning conformation control, charge microenvironment tuning and network topology engineering. In this architecture, a robust PAM skeleton provides mechanical support, whereas CMCS supplies percolated conduction domains and Zn^2+^-coordinating carboxylates. Introducing tB further suppresses amino protonation and increases carboxylate dissociation, raising local negative charge density to exclude polyiodides while creating low-barrier pathways for Zn^2+^ migration. The mildly alkaline environment also stretches CMCS chains and strengthens dynamic hydrogen-bonding with PAM, producing a topologically uniform interface that homogenizes Zn deposition. Concurrently, strong water binding within the network lowers free water activity, mitigating hydrogen evolution and corrosion without sacrificing conductivity. Consequently, the Zn symmetric cell achieved dendrite-free cycling for over 3500 h at 1 mA cm^−2^/1 mAh cm^−2^, and the Zn–I_2_ full cell retained 85.3% of its capacity after 20,000 cycles at 10 C rate. Overall, this work provides a hydrogel electrolyte comprehensive design framework from molecular engineering to mesoscale architecture, for advancing the development of high-performance aqueous Zn-based batteries.

## Supplementary Information

Below is the link to the electronic supplementary material.

Supplementary file1 (DOCX 24178 KB)
