# Supplementary material for: Ion-Sieving Dual-Network Hydrogel Electrolytes Couple Accelerated Ion Transport with Iodide Shuttle Suppression in Aqueous Zn–I2 Batteries
Source: Nanomicro Lett. 2026 May 11;18:366. doi: 10.1007/s40820-026-02216-6 (PMC13161454; doi:10.1007/s40820-026-02216-6)
Supplement: Supplementary file 1 — Supplementary file1 (DOCX 24178 KB) [file 40820_2026_2216_MOESM1_ESM.docx]

Supporting Information for

**Ion-Sieving Dual-Network Hydrogel Electrolytes Couple Accelerated Ion Transport with Iodide-Shuttle Suppression in Aqueous Zn-I_2_ Batteries**

Ming Chen^1^, Jia Cheng^1^, Yixin Zhao^1^, Wei Fu^1^, Wen Li^1, 2^*, Yunhai Zhu^3, 4^*, and Fanlu Meng^1, 2, 3^*

^1^ School of Materials Science and Engineering, Ocean University of China, Qingdao 266404, P. R. China

^2^ Key Laboratory of Marine Equipment Materials and Protection of Shandong Province, Qingdao 266404, P. R. China

^3^ Aerospace Special Power Source Technology Innovation Center, Tianfu Jiangxi Laboratory, Chengdu 641419, P. R. China

^4^ State Key Laboratory of New Textile Materials and Advanced Processing Technologies, Wuhan Textile University, Wuhan 430200, China

*Corresponding authors. E-mail: [liwen3710@ouc.edu.cn](mailto:liwen3710@ouc.edu.cn) (Wen Li); [yhzhu@wtu.edu.cn](mailto:yhzhu@wtu.edu.cn) (Yunhai Zhu); [mengfanlu@ouc.edu.cn](mailto:mengfanlu@ouc.edu.cn) (Fanlu Meng)

**Supplementary Note**

In ZnSO_4_ aqueous solution (pH≈5-6), the amino groups (pKa≈6.5) on CMCS are readily protonated to -NH_3_^+^, while the carboxyl groups (pKa≈4.5-5.0) are partially dissociated into -COO^-^. Upon the addition of tert-butylamine (pKa≈10.7), its stronger basicity compared to the amino groups of CMCS enables it to preferentially capture free H^+^ in the solution, leading to an increase in the local pH. According to acid-base equilibrium principles, the presence of tB shifts the equilibrium toward the deprotonated state, thereby reducing the proportion of protonated -NH_3_^+^ groups.

$$-{NH}_{3}^{+}\leftrightarrow{-NH}_{2}+H^{+}$$

In addition, the increase in local pH shifts the equilibrium toward dissociation, thereby promoting the formation of -COO^-^ groups.

$$-COOH\leftrightarrow{-COO}^{-}+H^{+}$$

Meanwhile, the reduction of -NH_3_^+^ groups weaken the electrostatic attraction between -NH_3_^+^ and -COO^-^, thereby releasing previously associated -COO^-^ groups.

In summary, tB establishes a locally weakly alkaline microenvironment within the gel network, thereby suppressing the protonation of -NH_2_ groups, eliminating positive charge centers, promoting the dissociation of -COOH groups, and increasing the density of negatively charged sites. The elimination of electrostatic attraction between -NH_3_^+^ and -COO^-^ promotes the extension of CMCS chains and exposes additional -COO^-^ groups. These synergistic effects collectively enable precise regulation of charge polarity and provide a molecular basis for subsequent rapid Zn^2+^ transport and electrostatic repulsion of polyiodide species.

**Supplementary Figures**


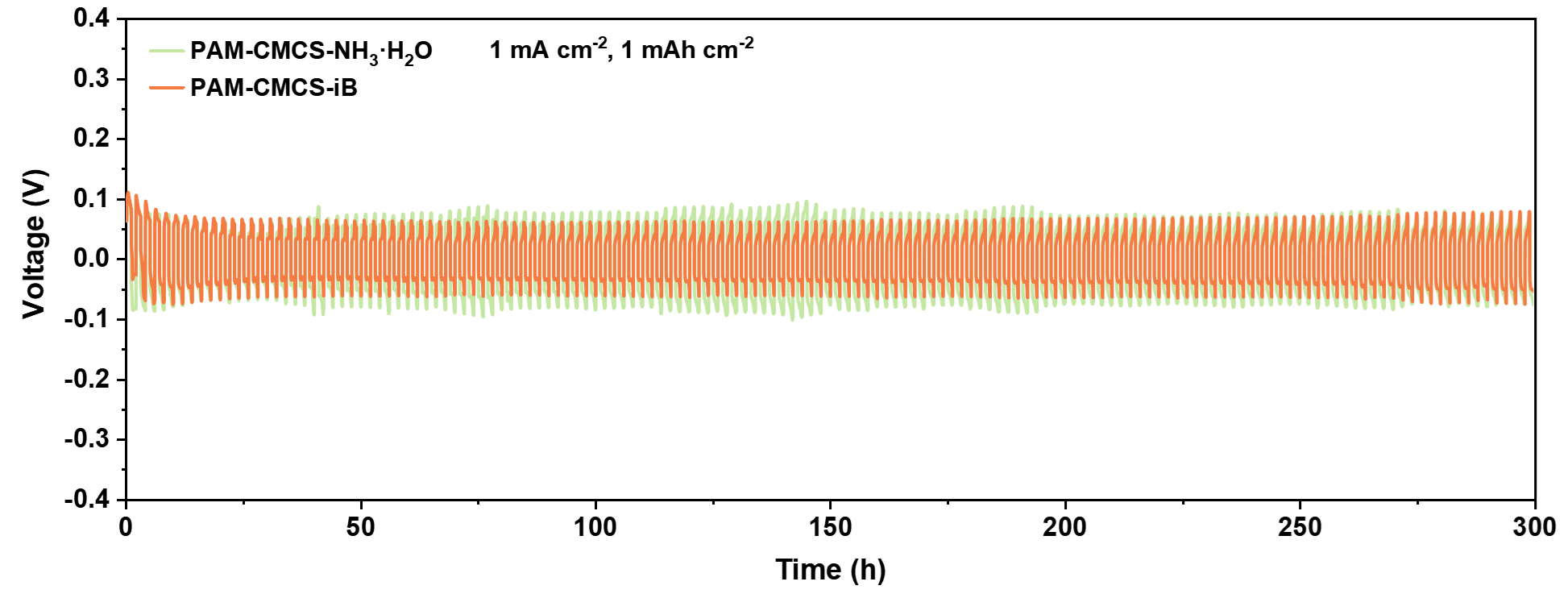


**Fig. S1** Cyclic performance of Zn//Zn symmetric cells assembled with different hydrogel electrolytes at current density of 1 mA cm^-2^ and area capacity matching of 1 mAh cm^-2^

To clarify the effect of alkyl groups on the hydrogel performance, ammonia and isobutylamine are used to replace tert-butylamine, and corresponding symmetric cells are assembled to evaluate cycling performance. In the PAM-CMCS-NH_3_·H_2_O hydrogel, the effect of alkyl groups is eliminated, whereas in the PAM-CMCS-iB hydrogel, an alternative alkyl structure is introduced. The results show that the cycling performance of the assembled symmetric cells is comparable to that of PAM-CMCS-tB, suggesting that the enhanced performance primarily originates from the weakly alkaline environment established by tert-butylamine, which promotes further deprotonation of amino groups and dissociation of carboxyl groups.


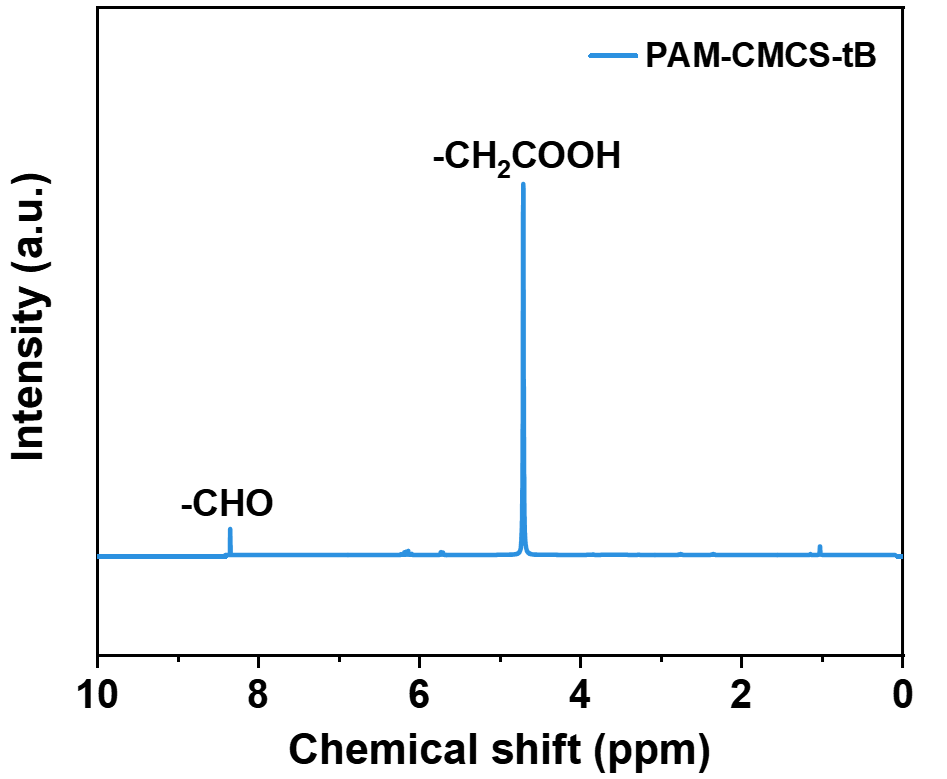


**Fig. S2** ^1^H NMR spectra of PAM-CMCS-tB hydrogel

For the quantitative analysis of carboxyl content in PAM-CMCS-tB hydrogel, sodium formate is used as the internal standard, with its characteristic -CHO peak serving as the reference, and the carboxyl content is calculated using the following formula:

$${Content}_{-COOH}=\frac{\frac{I_{CMCS}}{N_{CMCS}}}{\frac{I_{HCOONa}}{N_{HCOONa}}}\times n_{HCOONa}/m_{gel}$$

Here, ***I*** is the integrated area of the target peak, ***N*** is the number of protons in the target group, ***n*** denotes the amount (in moles) of the internal standard in the sample, and ***m*** is the mass of the hydrogel sample.


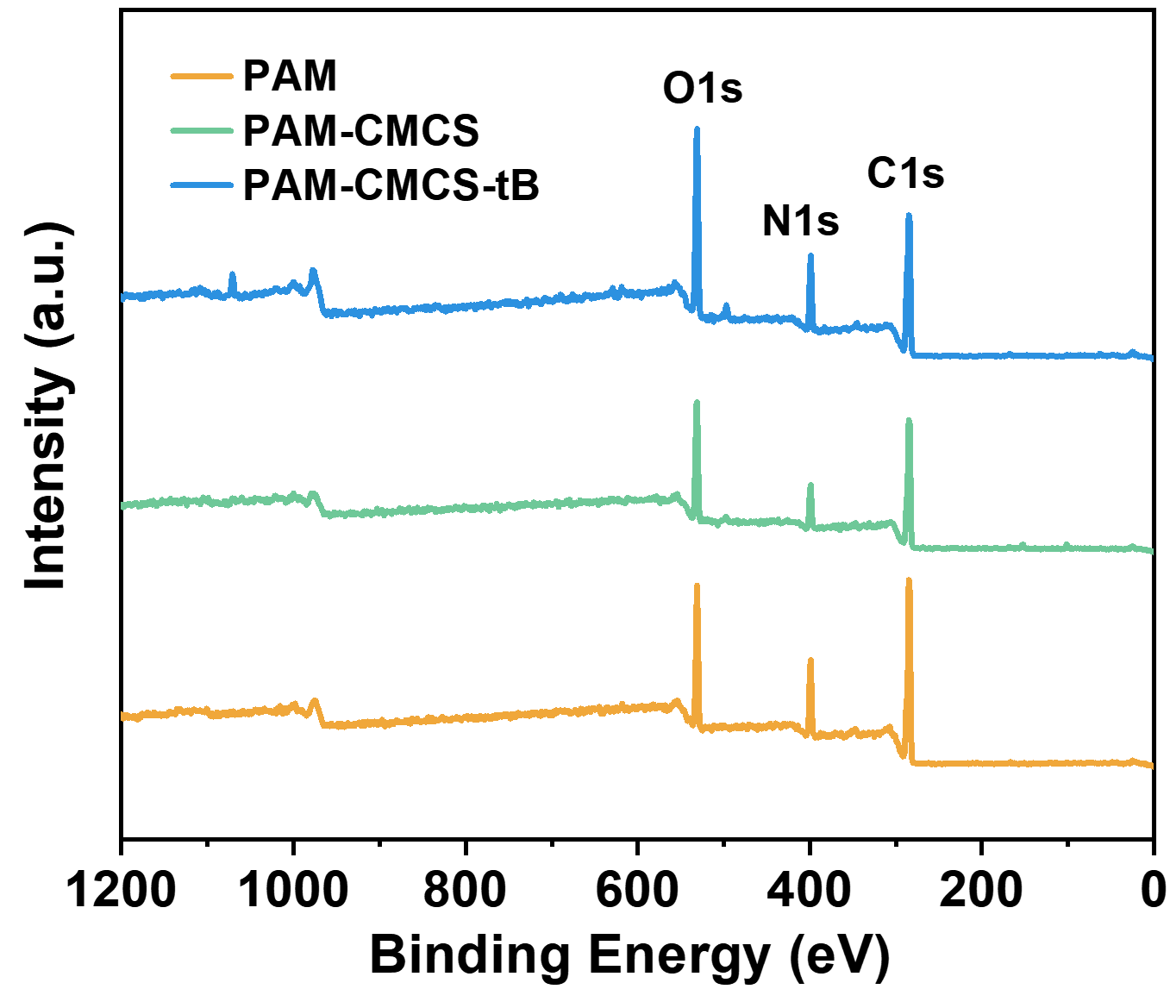


**Fig. S3** XPS spectra of all elements


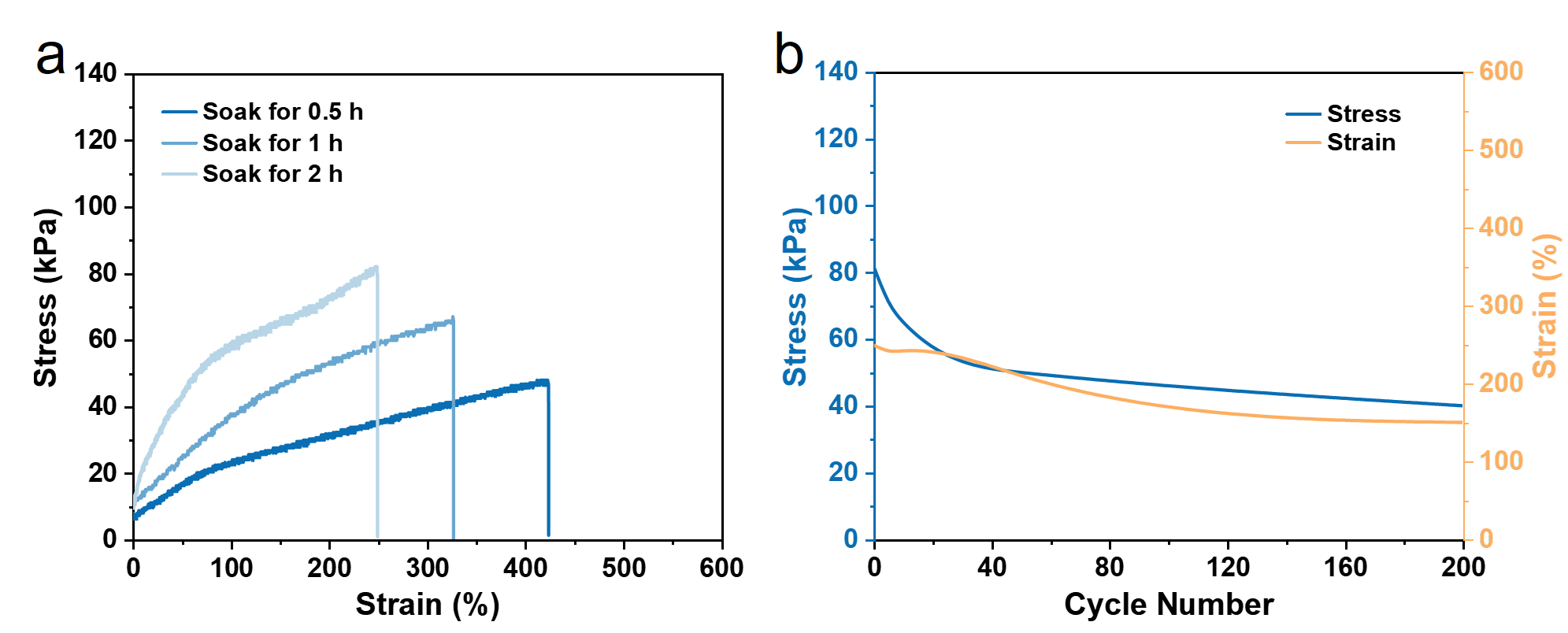


**Fig. S4** **a** Stress-strain curves of the PAM-CMCS-tB hydrogel after immersion in 2 M ZnSO_4_ for different times. **b** The stress-strain behavior of the PAM-CMCS-tB hydrogel electrolyte evolves with increasing cycle number


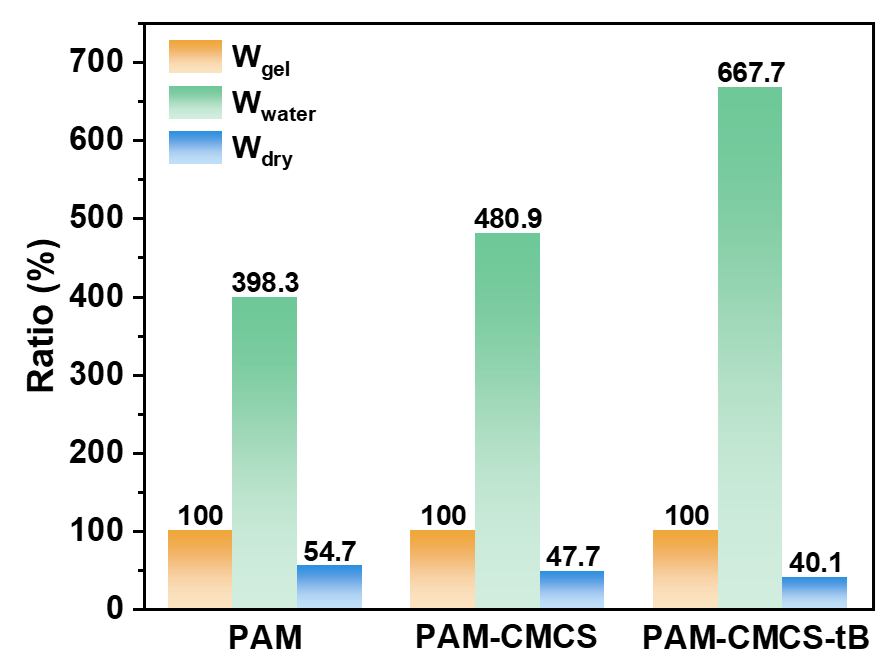


**Fig. S5** Composition changes of different gel electrolytes in deionized water


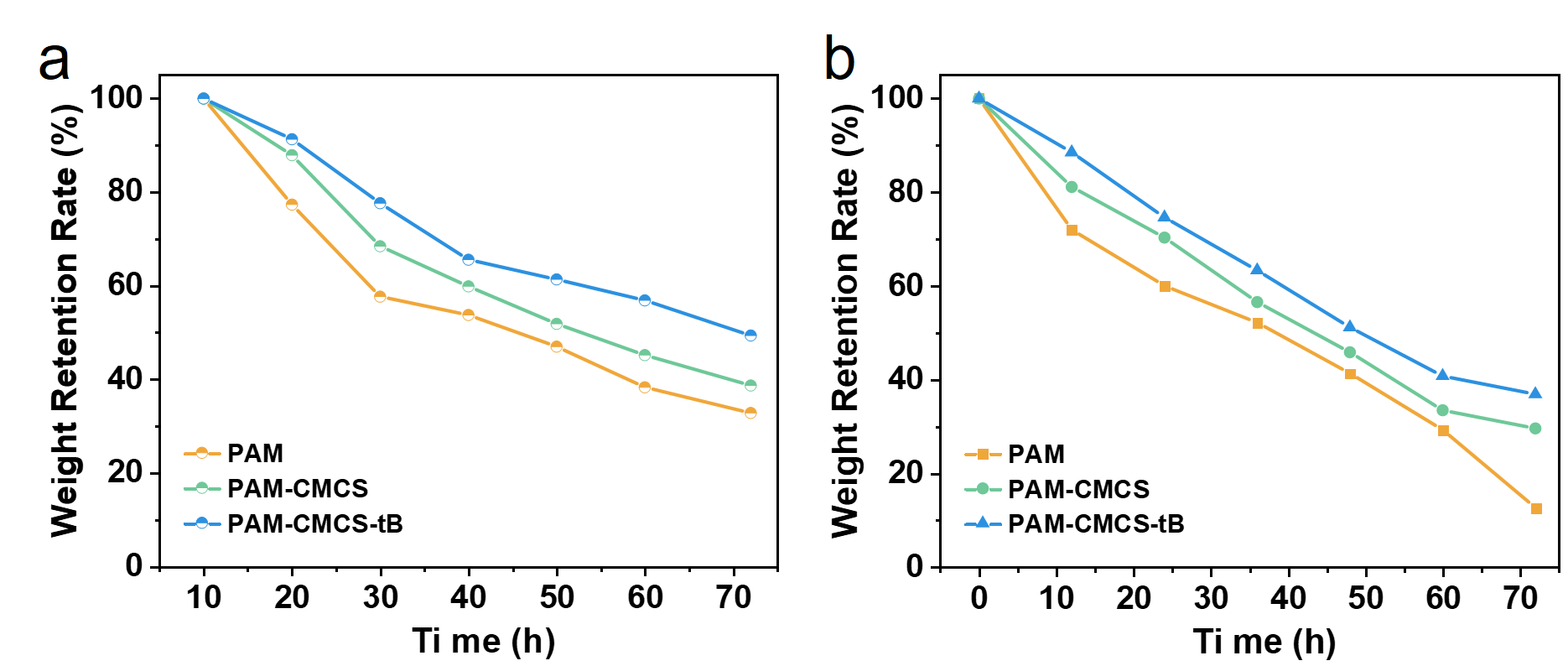


**Fig. S6** Comparison of water retention capabilities of various hydrogels in different media at 25 °C. **a** 2 M ZnSO_4_, **b** deionized water


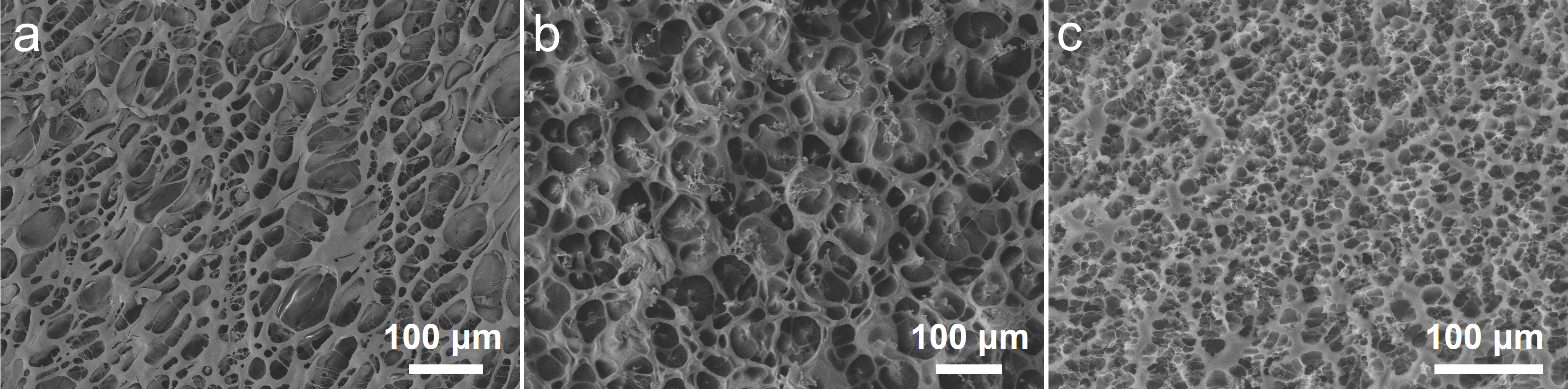


**Fig. S7** SEM images of different hydrogels. **a** PAM, **b** PAM-CMCS, **c** PAM-CMCS-tB


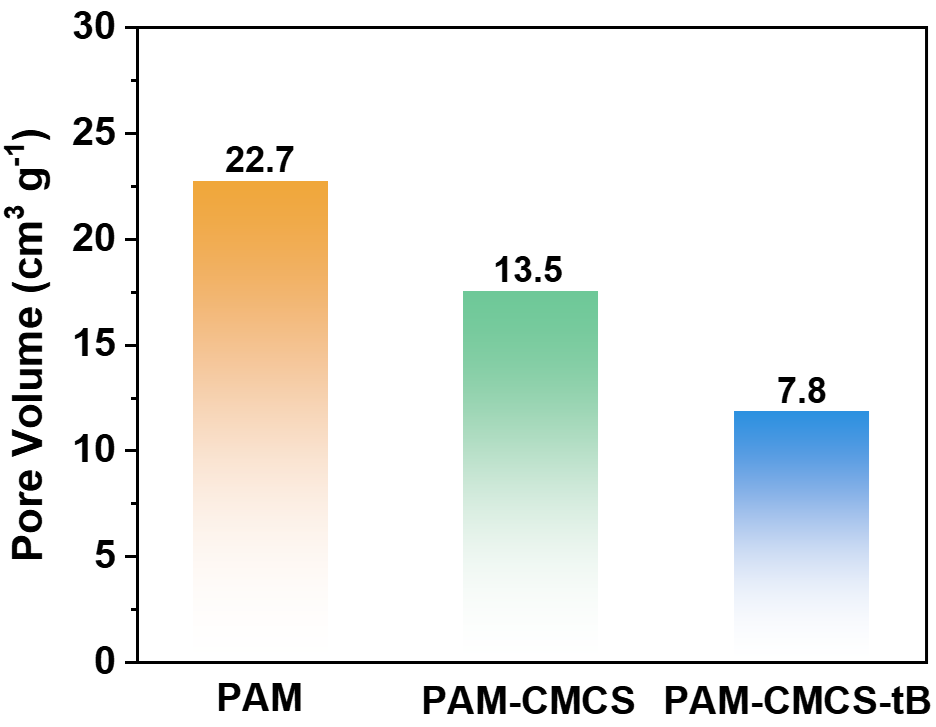


**Fig. S8** Pore volume of different hydrogels


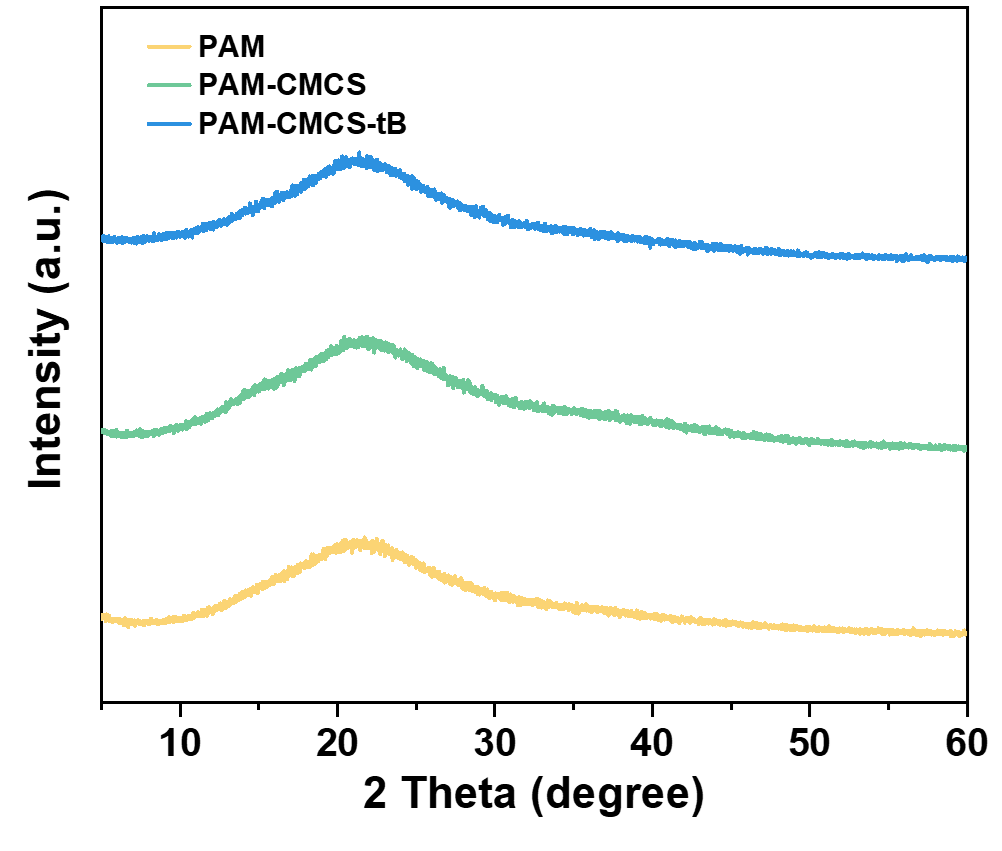


**Fig. S9** XRD patterns of different hydrogels


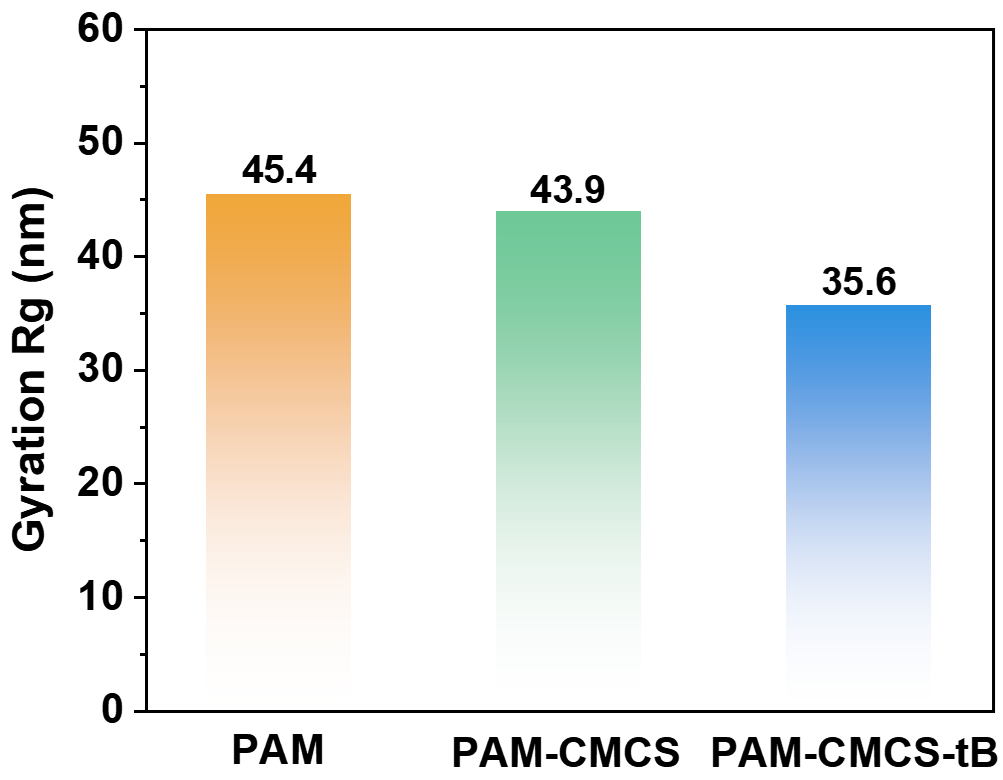


**Fig.** **S10** The radius of gyration (Rg) of the hydrogel electrolyte, as determined by SAXS


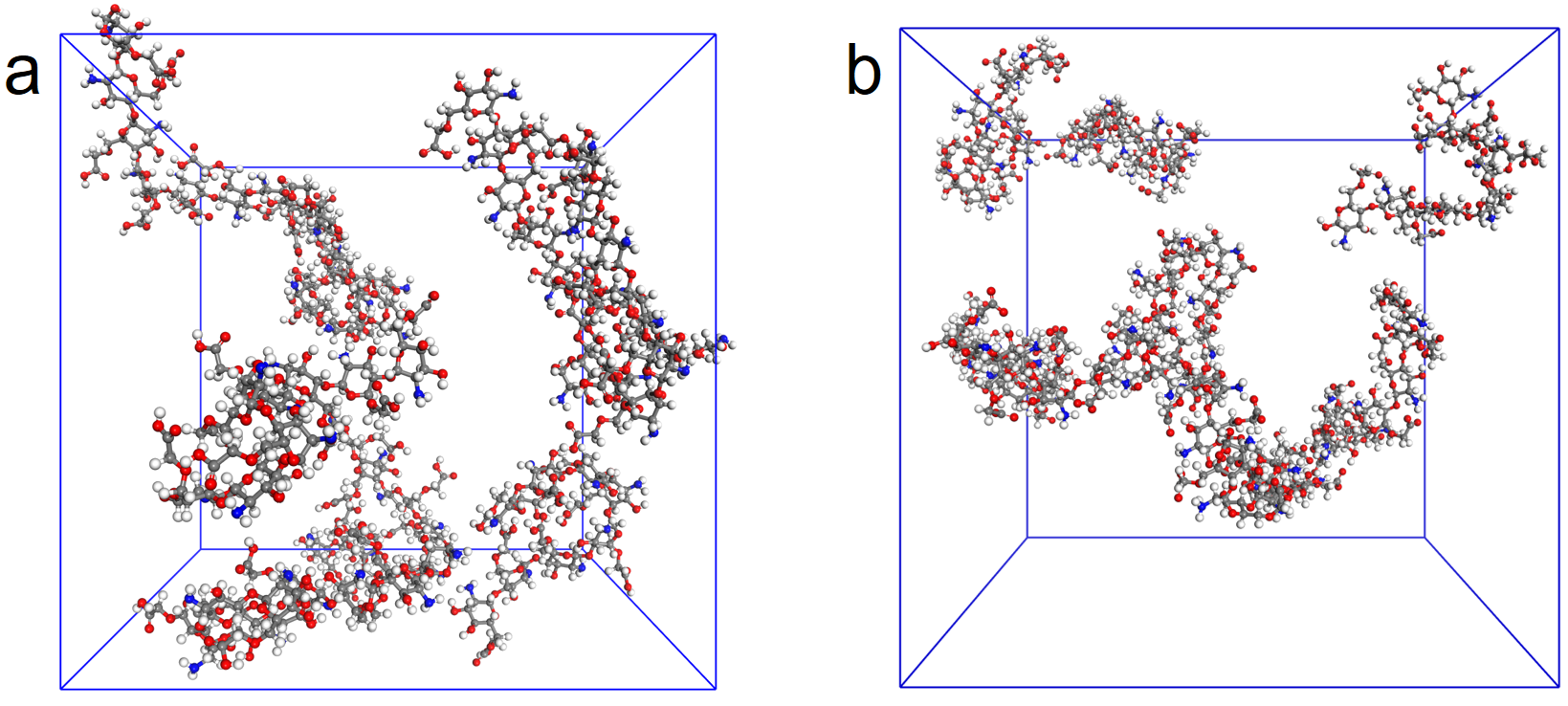


**Fig. S11** Molecular dynamics simulations showing the structural configuration of the **a** PAM-CMCS-tB and **b** PAM-CMCS hydrogel network. (PAM in the system has been hidden.)


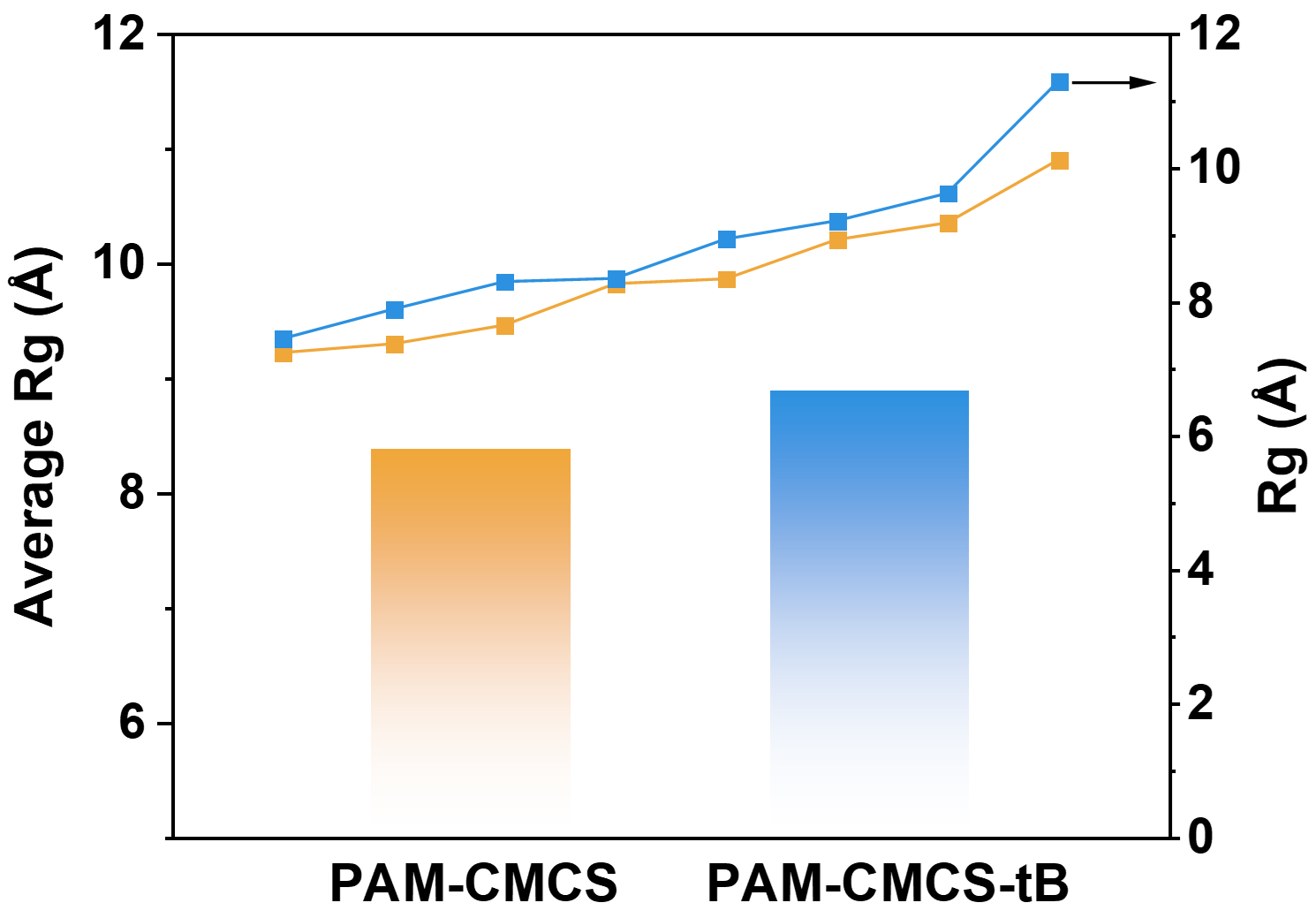


**Fig. S12** Molecular dynamics simulation calculates the radius of rotation of CMCS chains in polymers


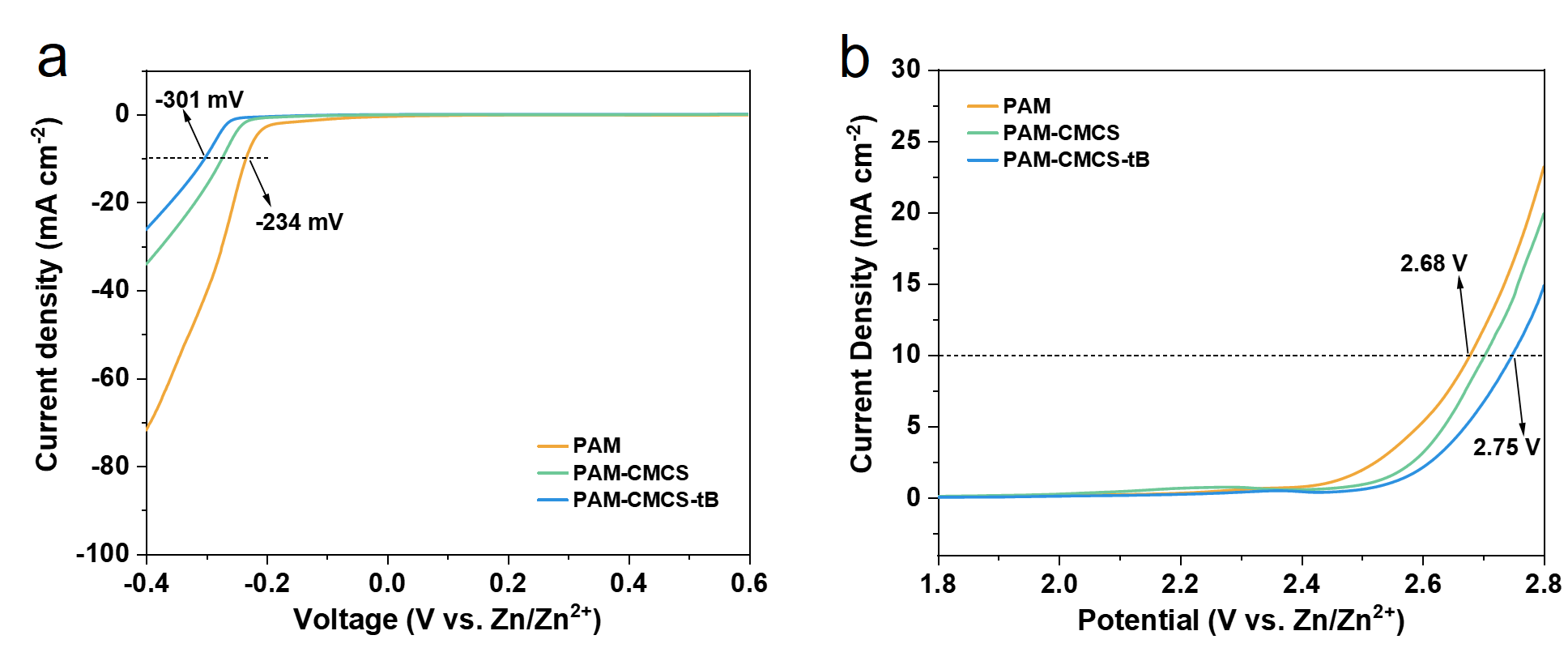


**Fig. S13** LSV curves for **a** HER and **b** OER


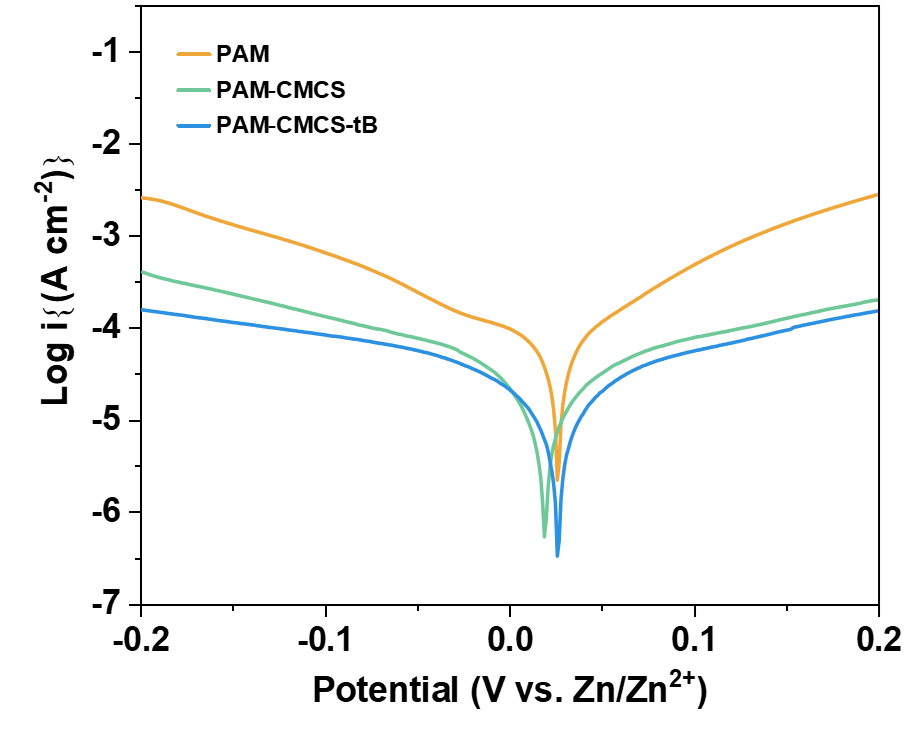


**Fig. S14** Tafel plots of Zn anodes in different hydrogel electrolytes


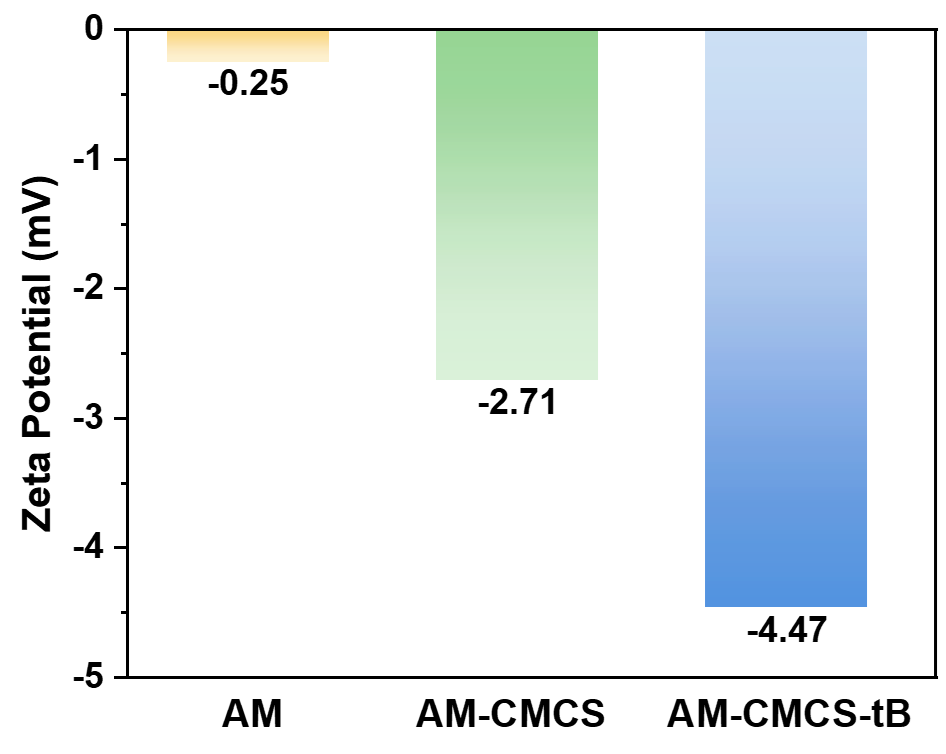


**Fig. S15** The zeta potential in different hydrogel systems


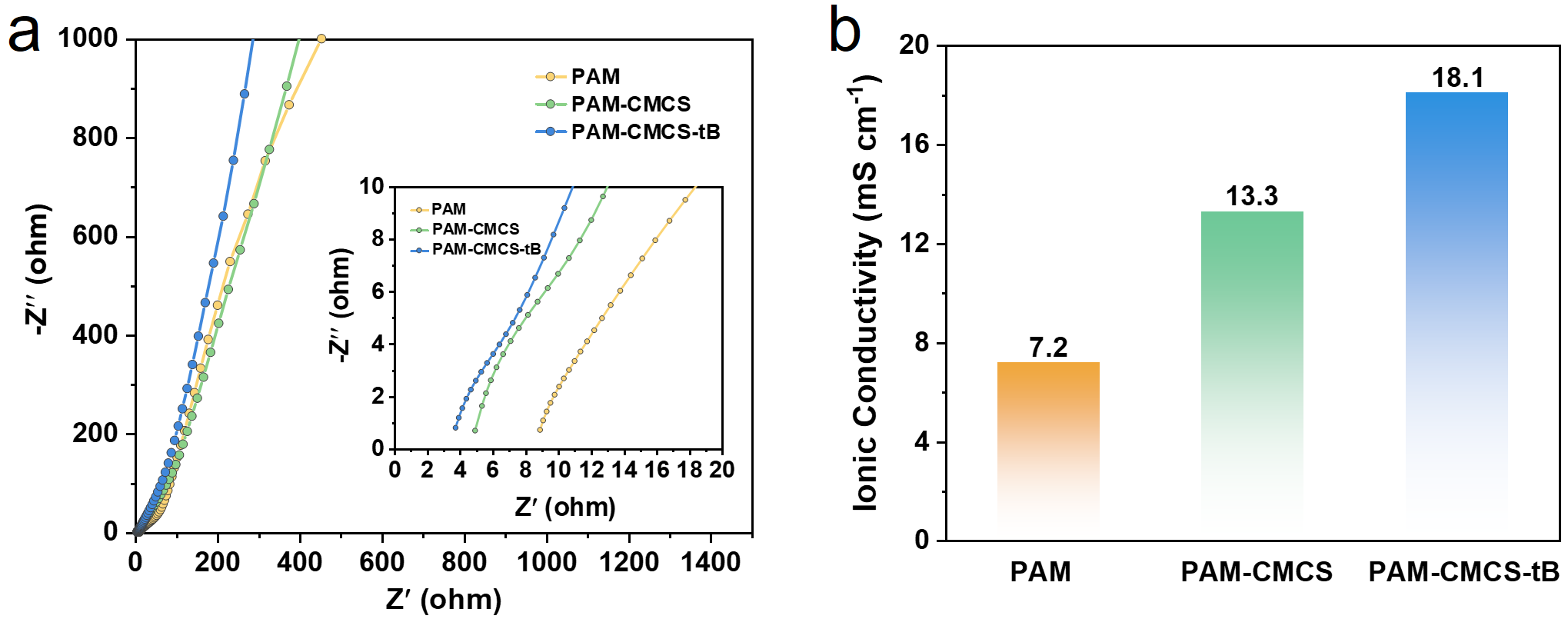


**Fig. S16** Stainless steel foil was used as working electrode and counter electrode, and symmetrical cells were assembled with different gel electrolytes to measure ionic conductivity. **a** Nyquist plots, **b** Ionic conductivity

The ionic conductivity (σ) of the hydrogel electrolyte was calculated using the equation:

$$\sigma=\frac{l}{RA}$$

where l is the thickness of the hydrogel electrolyte, R is the resistance obtained from the impedance spectrum of the symmetric cell, and A is the contact area of the electrode.
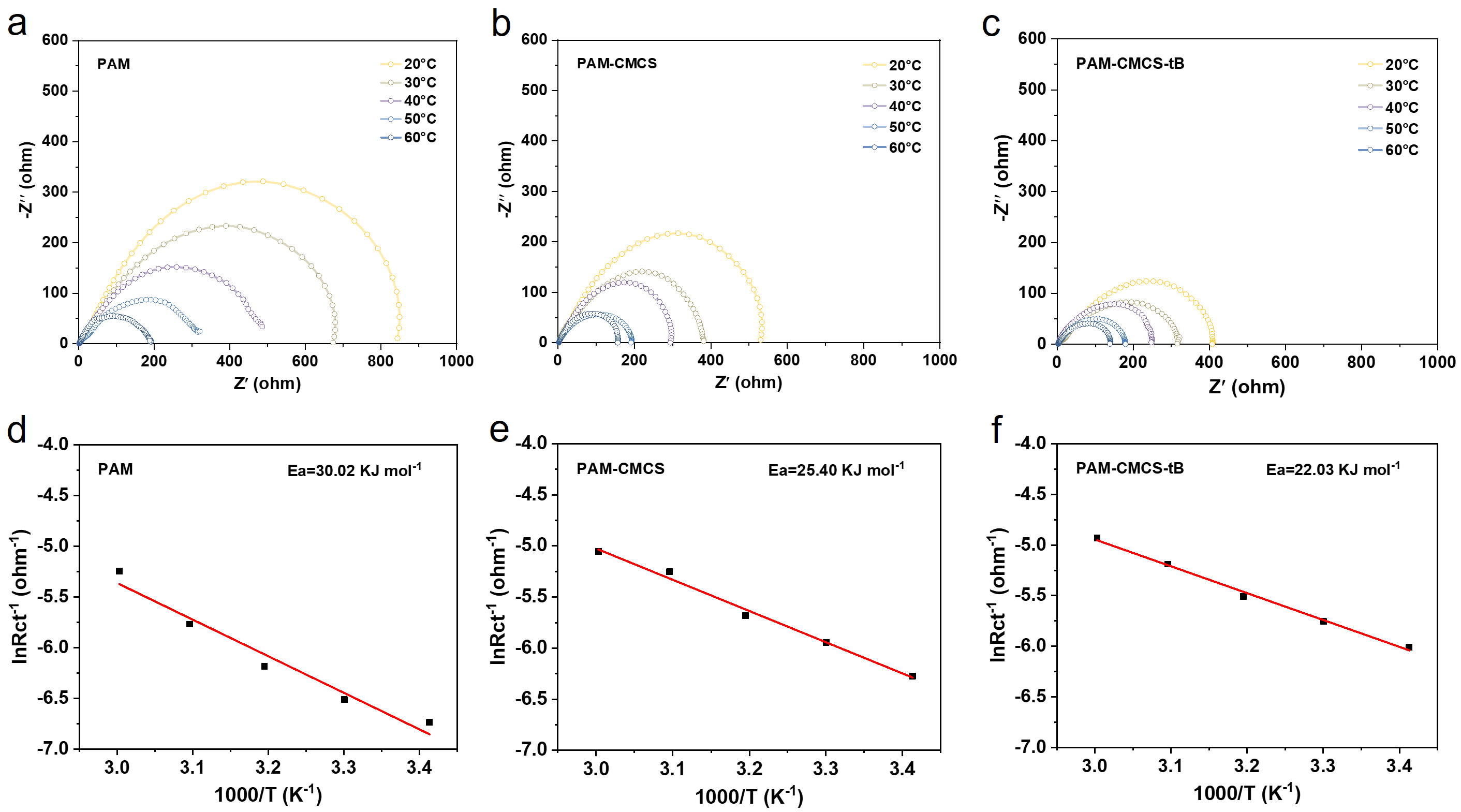


**Fig. S17** **a-c** Electrochemical impedance spectroscopy (EIS) spectra of Zn//Zn symmetric cells employing PAM, PAM-CMCS, and PAM-CMCS-tB hydrogel electrolytes at various temperatures, respectively. **d-f** Corresponding Arrhenius plots used to calculate the activation energies (Ea) for ion transport in each system

The activation energy was determined by fitting the impedance data to the Arrhenius equation:

$$\frac{1}{Rct}=Aexp\left( -\frac{Ea}{RT} \right)$$

where A denotes the pre-exponential factor, R is the universal gas constant, T is the absolute temperature, and Rct represents the charge-transfer resistance obtained from EIS measurements at various temperatures.


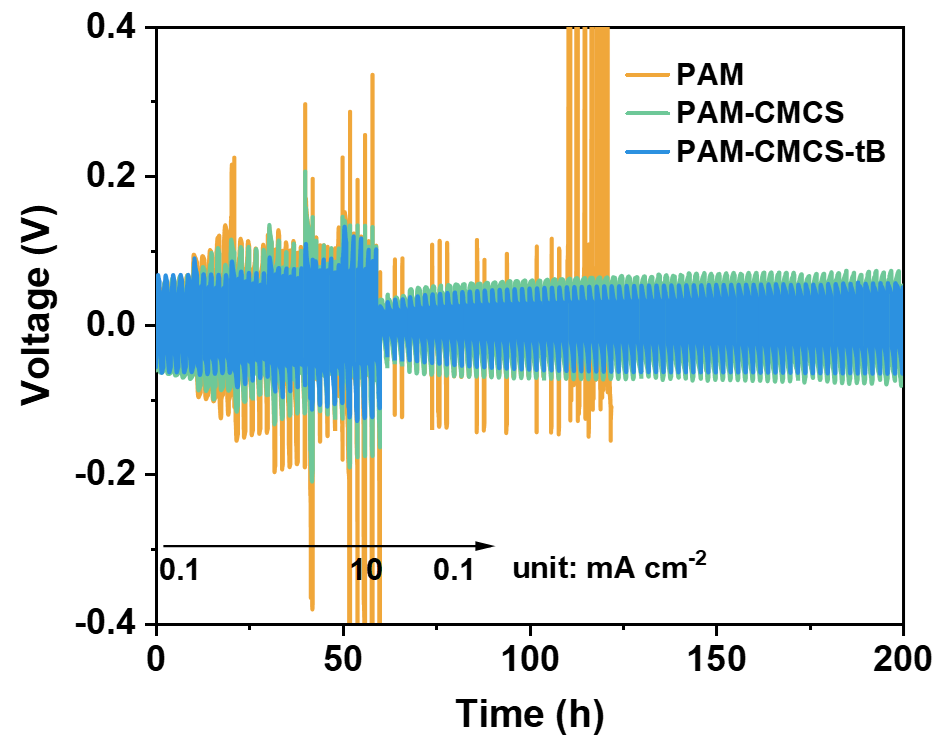


**Fig. S18** Zn deposition/stripping rate performance of Zn//Zn symmetric cells at different current densities


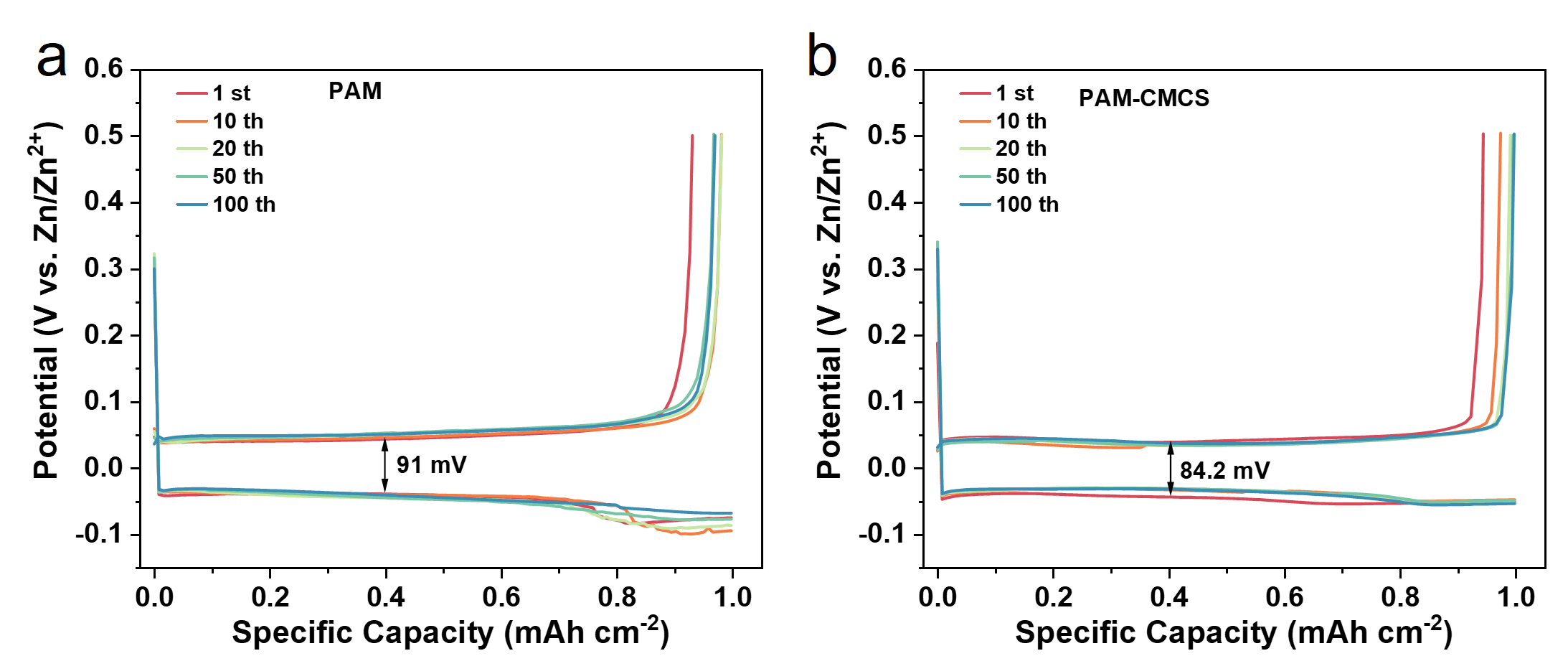


**Fig. S19** Zn deposition/stripping voltage profiles in half-cell tests. **a** PAM and **b** PAM-CMCS hydrogel electrolytes


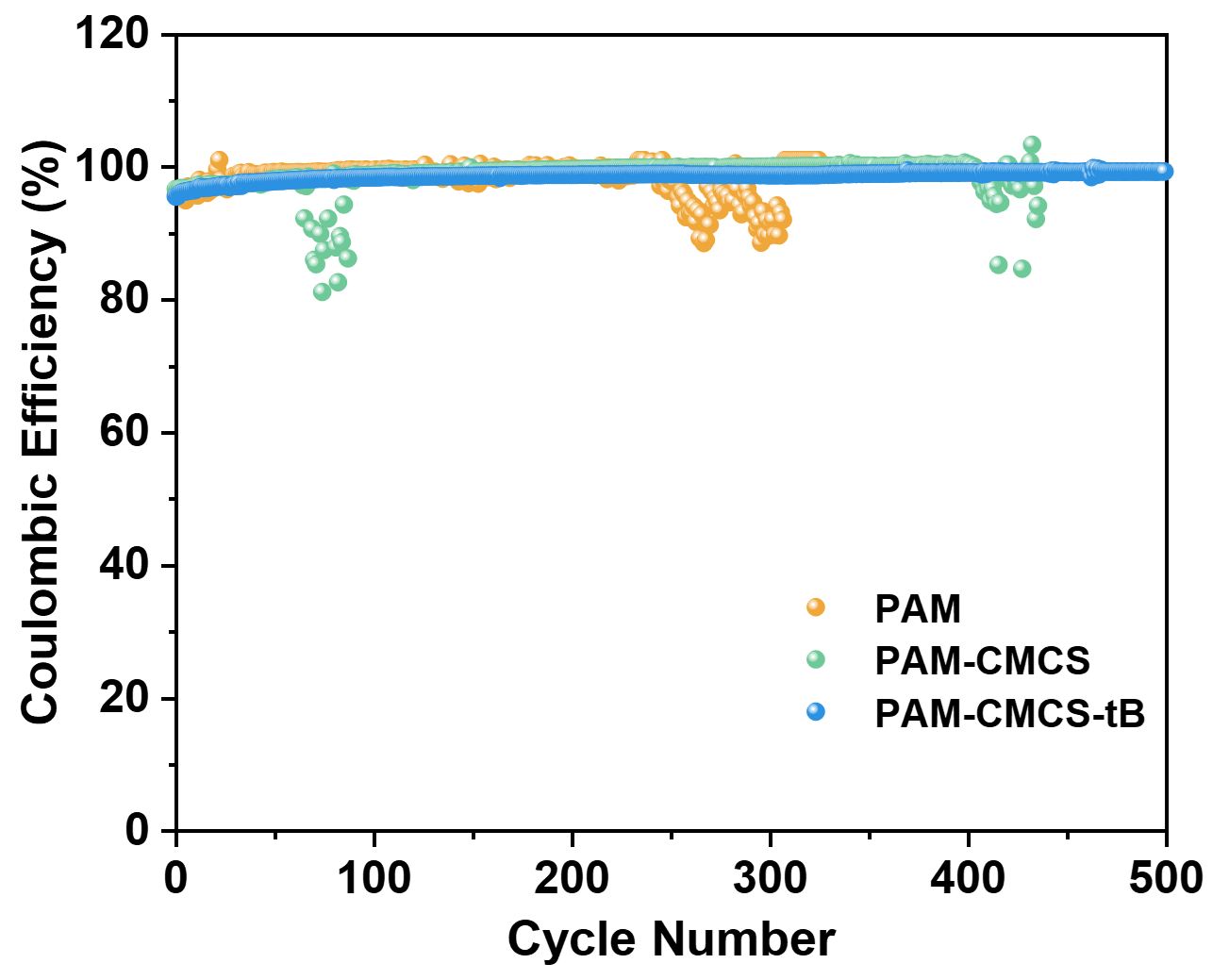


**Fig. S20** Coulombic efficiency cycling performance under half cell testing


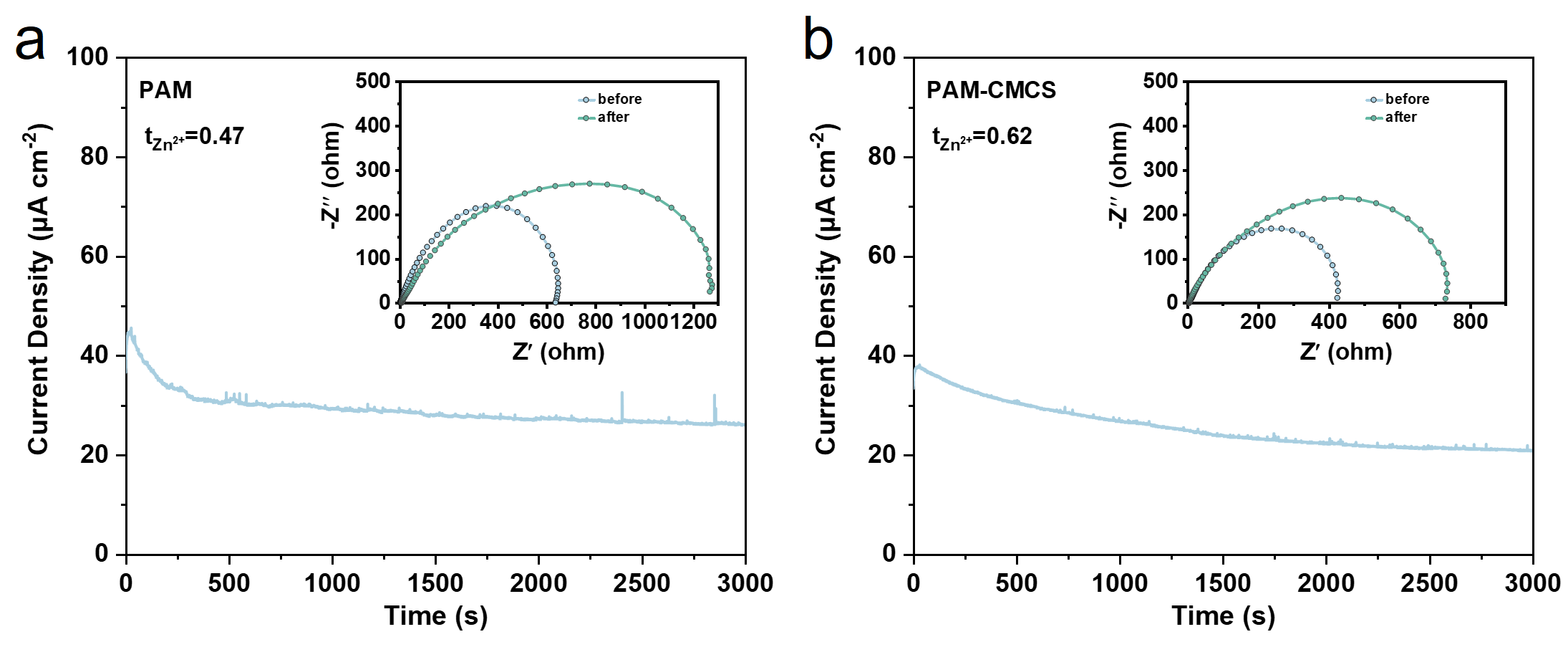


**Fig. S21** Chronoamperometric curves recorded at a constant potential of 20 mV, with the inset showing Nyquist plots obtained before and after polarization. **a** PAM and **b** PAM-CMCS hydrogel electrolytes

The Zn^2+^ transference number was calculated using the following equation:

$$t_{{Zn}^{2+}}=\frac{I_{S}\left( \Delta V-I_{0}R_{0} \right)}{I_{o}\left( \Delta V-I_{s}R_{s} \right)}$$

where ΔV denotes the applied potential difference; I_0_ and R_0_ are the initial current and interfacial resistance before polarization, and I_s_ and R_s_ represent the steady-state current and resistance after polarization.


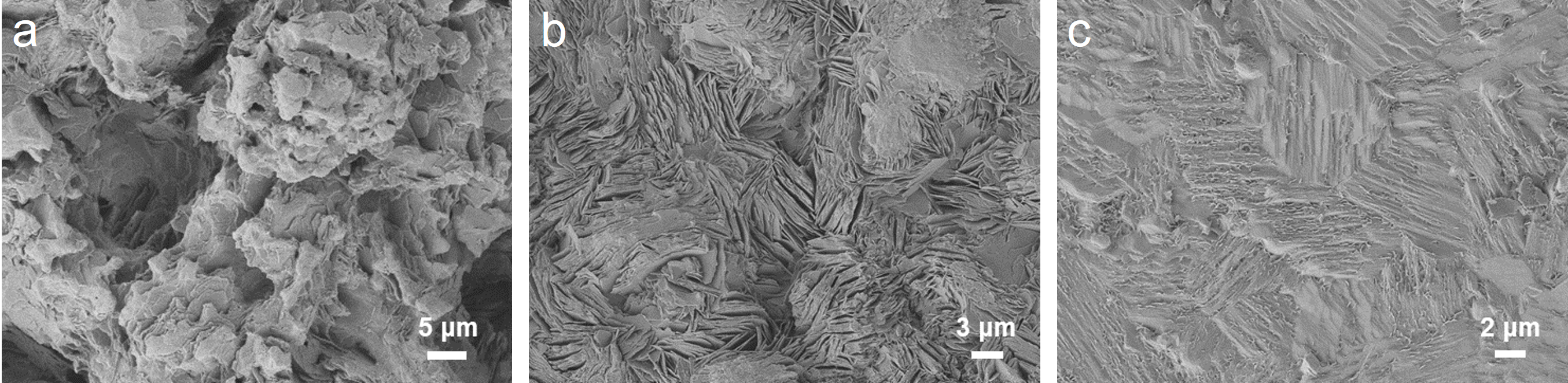


**Fig. S22** SEM images of Zn anode surfaces after 100 cycles of plating/stripping at 1 mAh cm^-2^ for **a** PAM, **b** PAM-CMCS, and **c** PAM-CMCS-tB hydrogel electrolytes

**
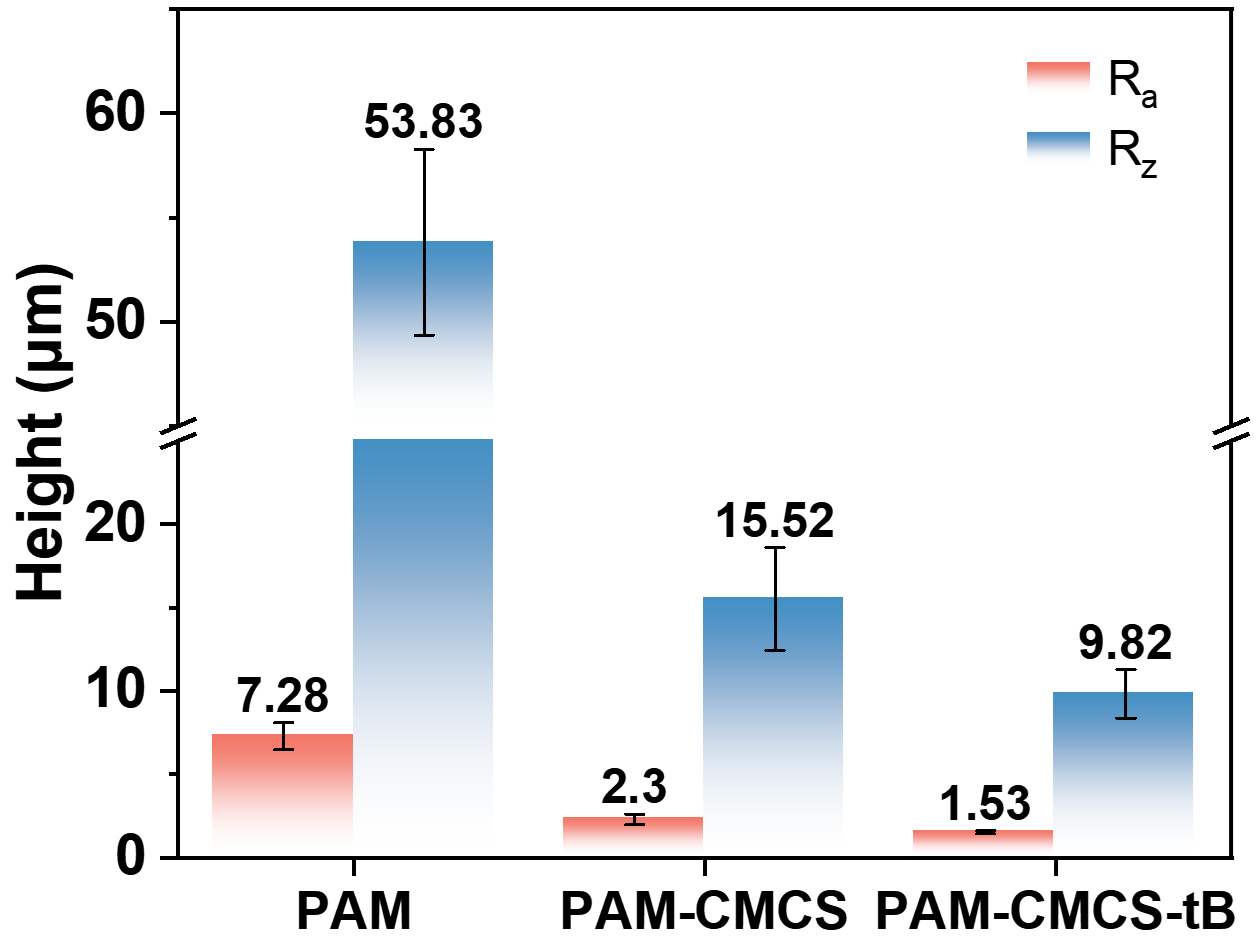
**

**Fig. S23** The average roughness (Ra) and maximum height (Rz) of the zinc electrode after cycling


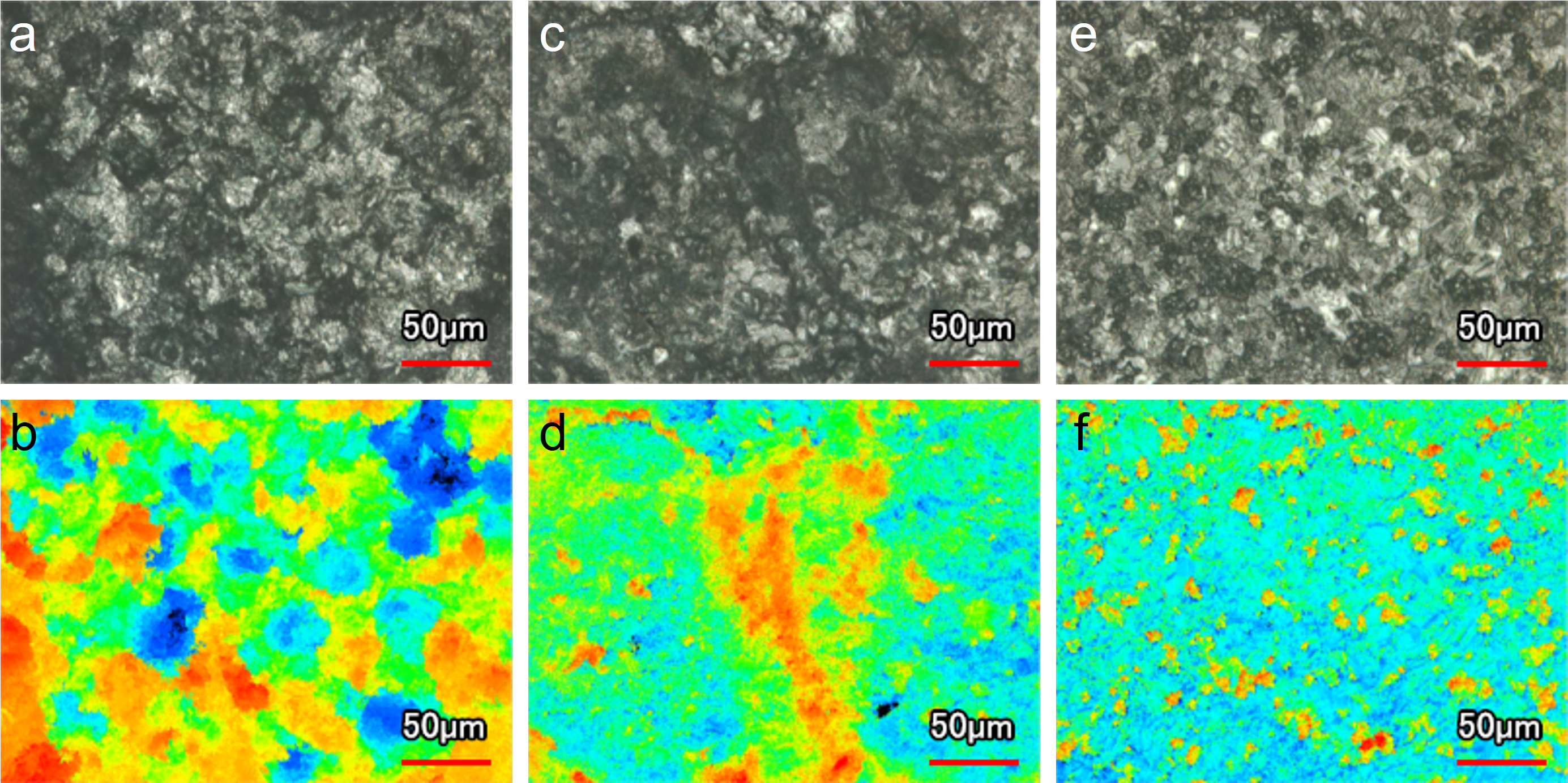


**Fig. S24** Images of the electrode surface under a laser confocal microscope after 100 cycles at 1 mA cm^-2^ and 1 mAh cm^-2^ for **a**, **b** PAM, **c**, **d** PAM-CMCS, and e, f) PAM-CMCS-tB hydrogel electrolytes

**
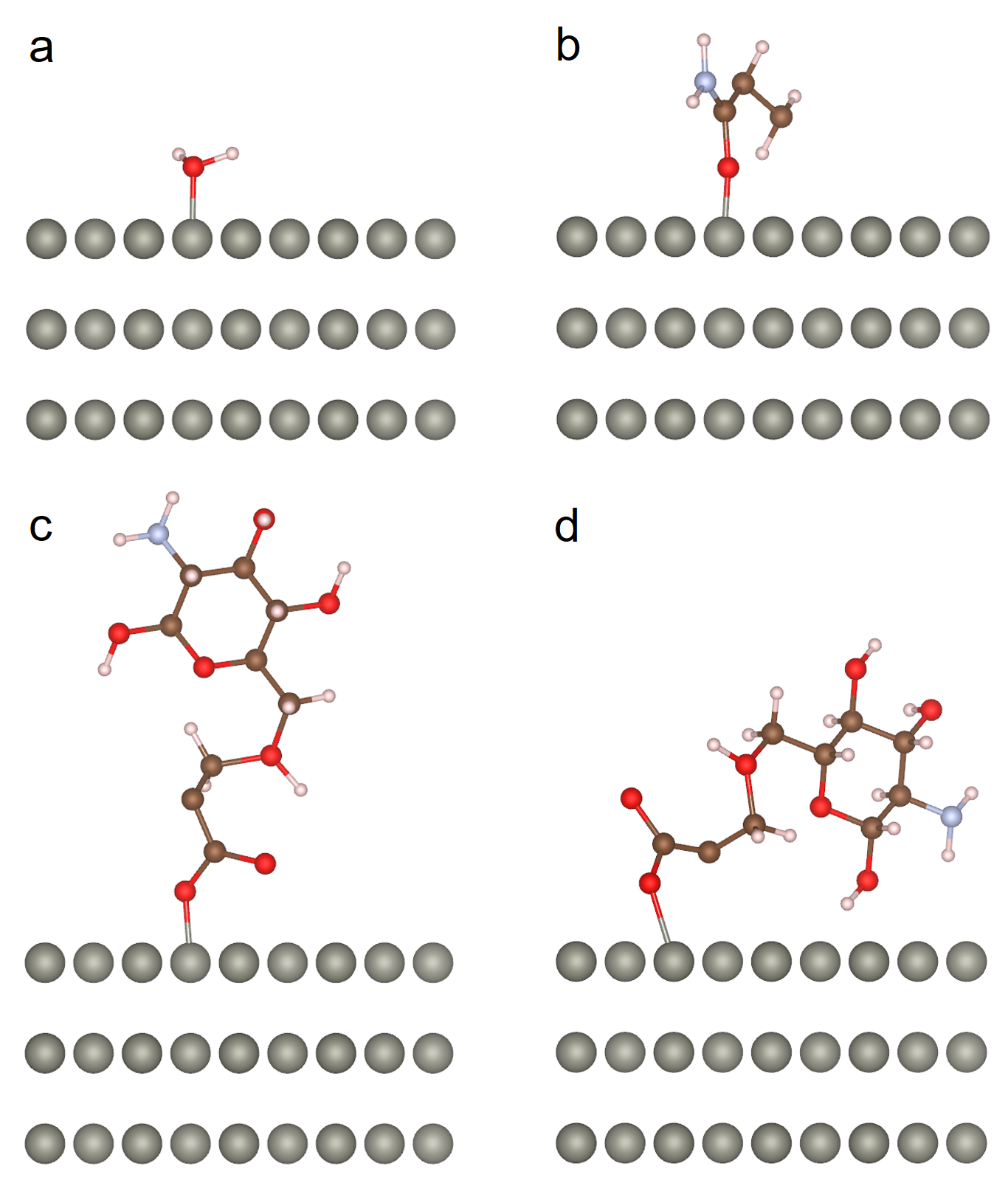
**

**Fig. S25** The adsorption configurations of different molecules on the Zn (002) surface are shown as follows: **a** H_2_O, **b** AM, **c** vertically oriented CMCS, and **d** horizontally oriented CMCS

**
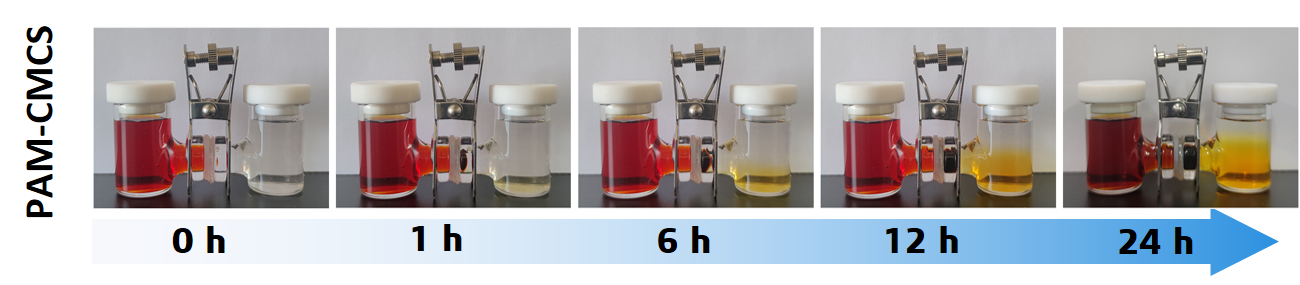
**

**Fig. S26** Optical images of the polyiodide ion diffusion in H-type cells separated by PAM-CMCS hydrogel


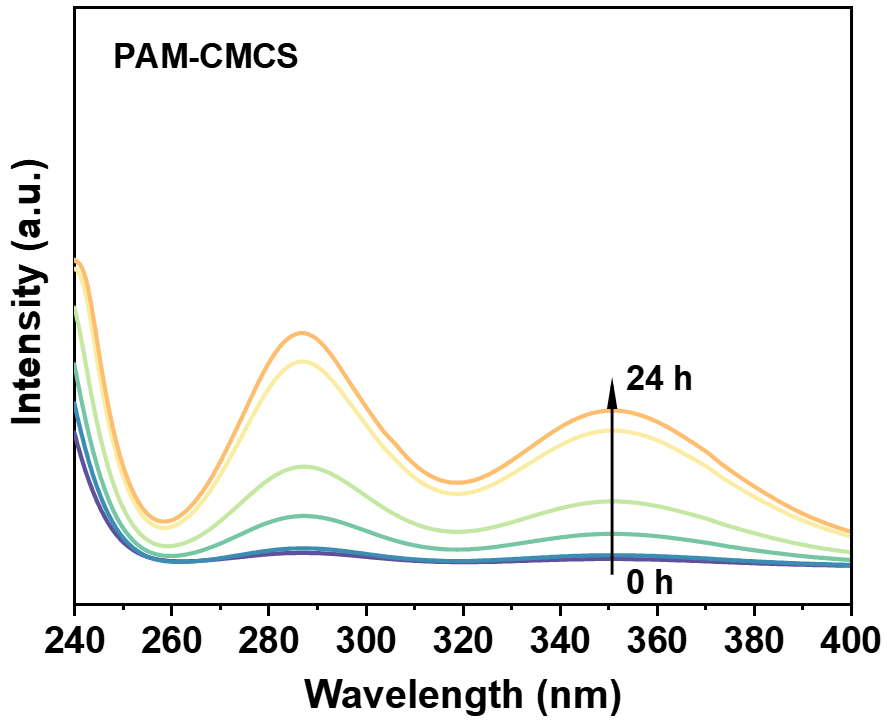


**Fig. S27** UV-vis absorption spectra of the solution in the right chamber at different time intervals using PAM-CMCS hydrogel


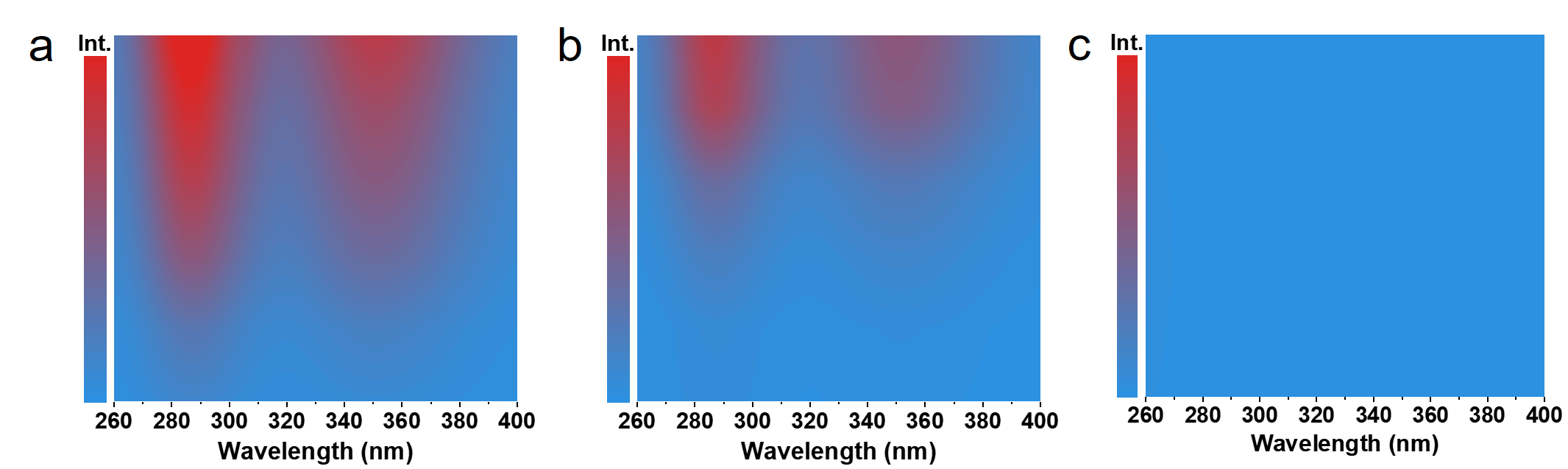


**Fig. S28** UV-vis absorption spectra of the solution in the right chamber of the H-type cell were collected at various time intervals for **a** the glass fiber membrane, **b** the PAM-CMCS hydrogel and **c** the PAM-CMCS-tB hydrogel


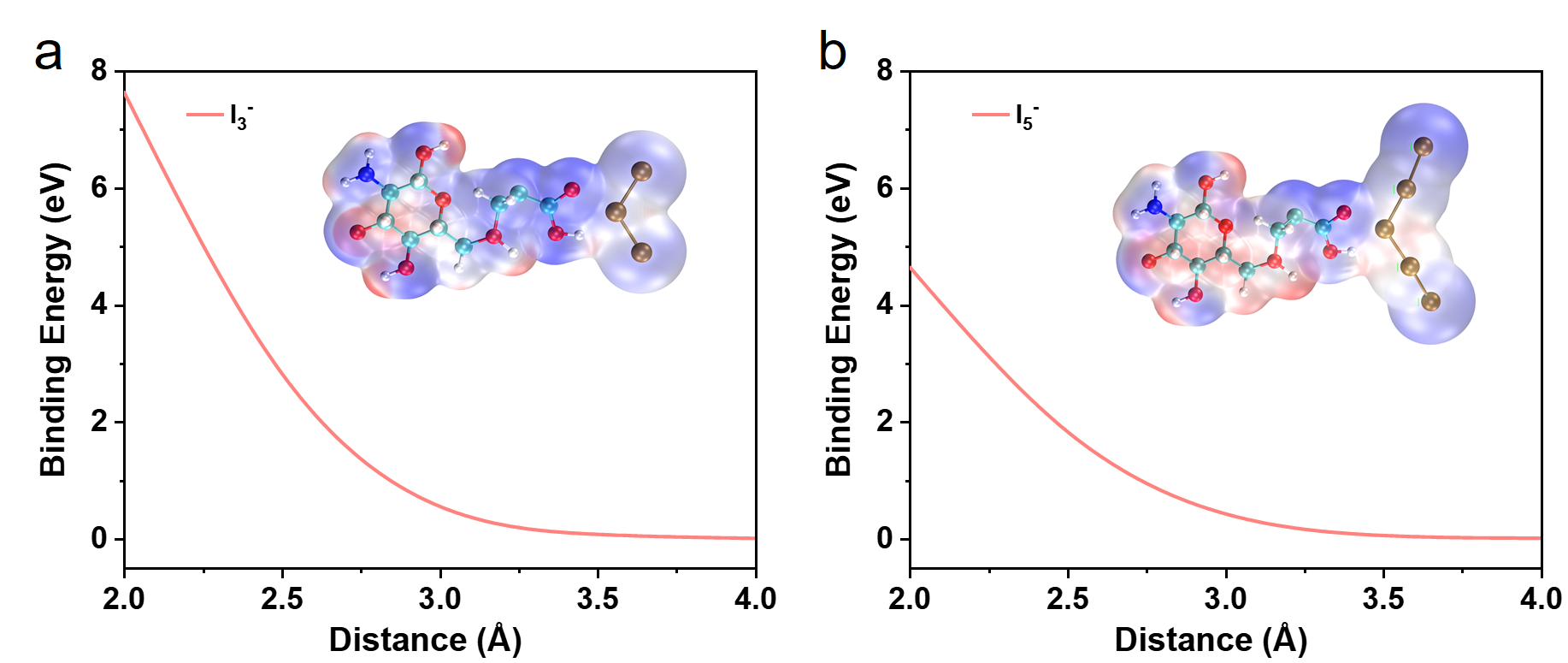


**Fig. S29** Calculated binding energies between polyiodide ions and functional groups on PAM-CMCS-tB polymer chains: **a** I_3_^-^ and **b** I_5_^-^

**
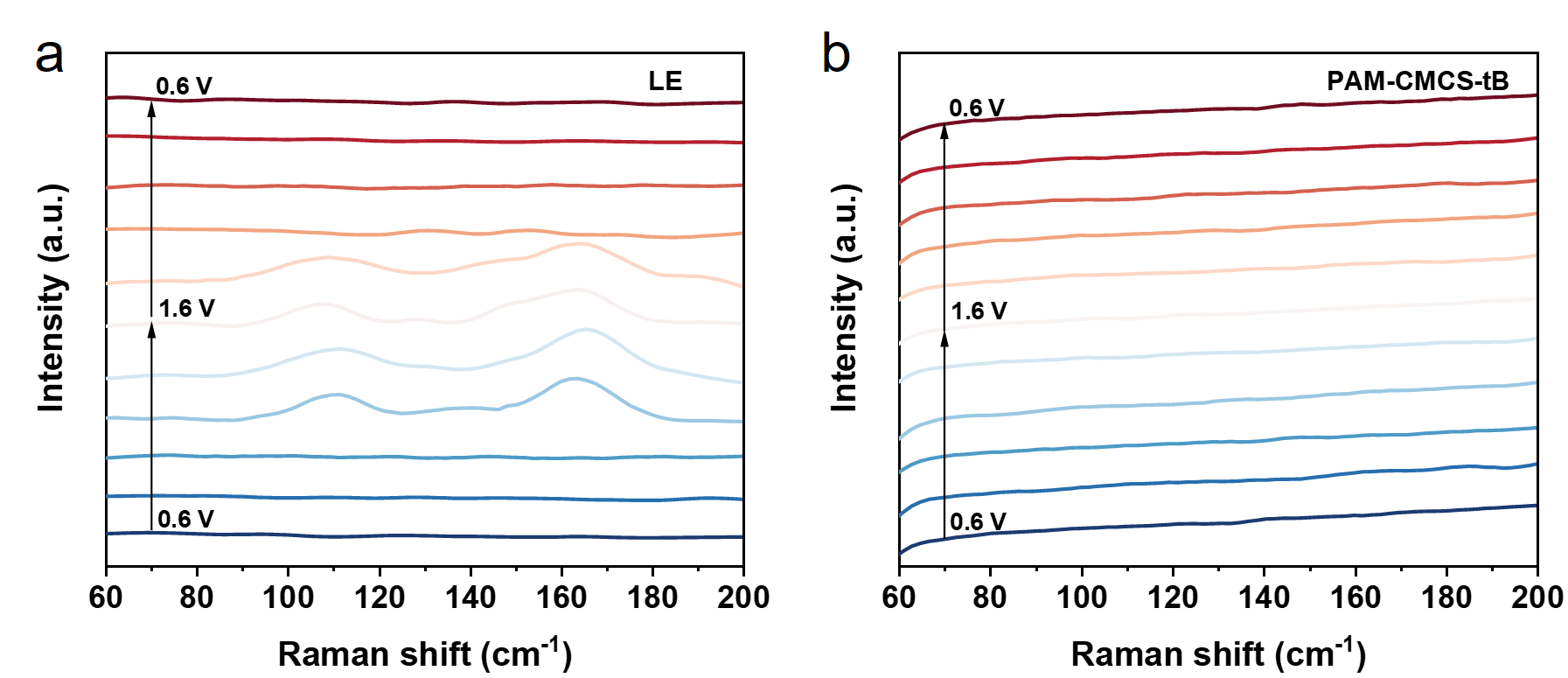
**

**Fig. S30** *In situ* Raman spectra of Zn-I_2_ cells were recorded at various voltages during a single charge-discharge cycle using **a** the liquid electrolyte and **b** the PAM-CMCS-tB hydrogel electrolyte


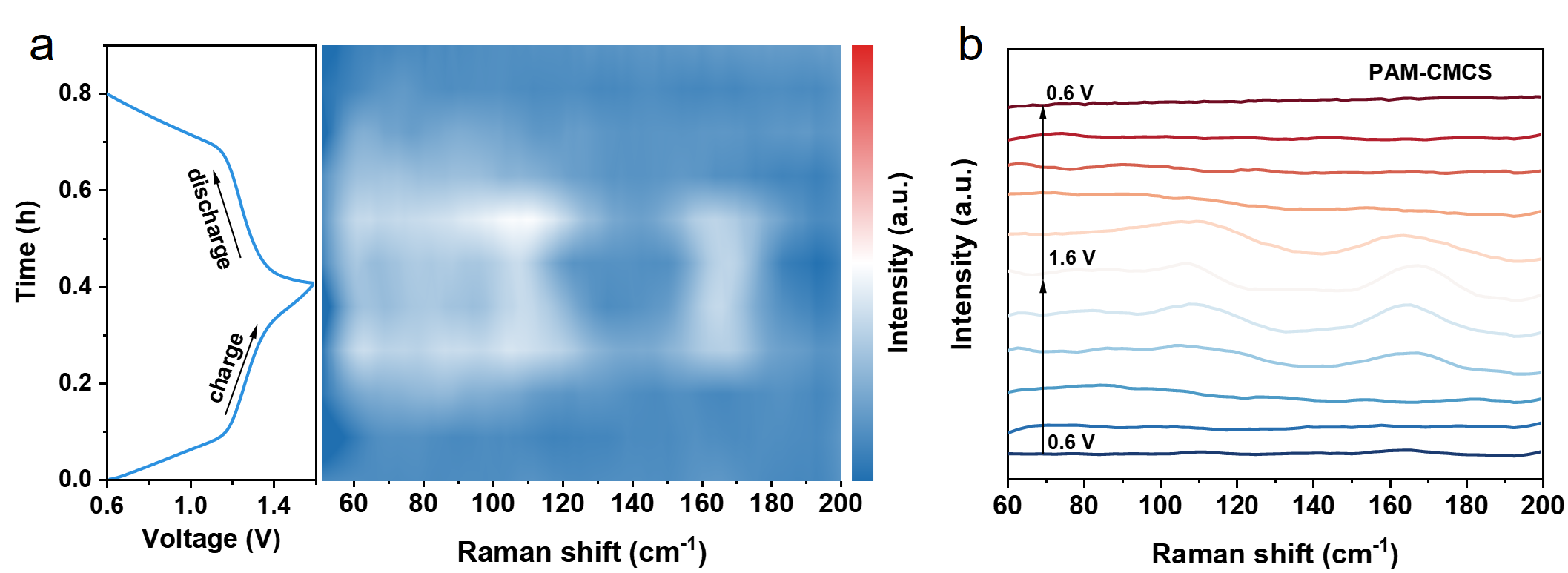


**Fig. S31 a, b** Voltage-time profiles and corresponding *in situ* Raman spectra of Zn-I_2_ batteries during one charge-discharge cycle using the PAM-CMCS hydrogel electrolyte


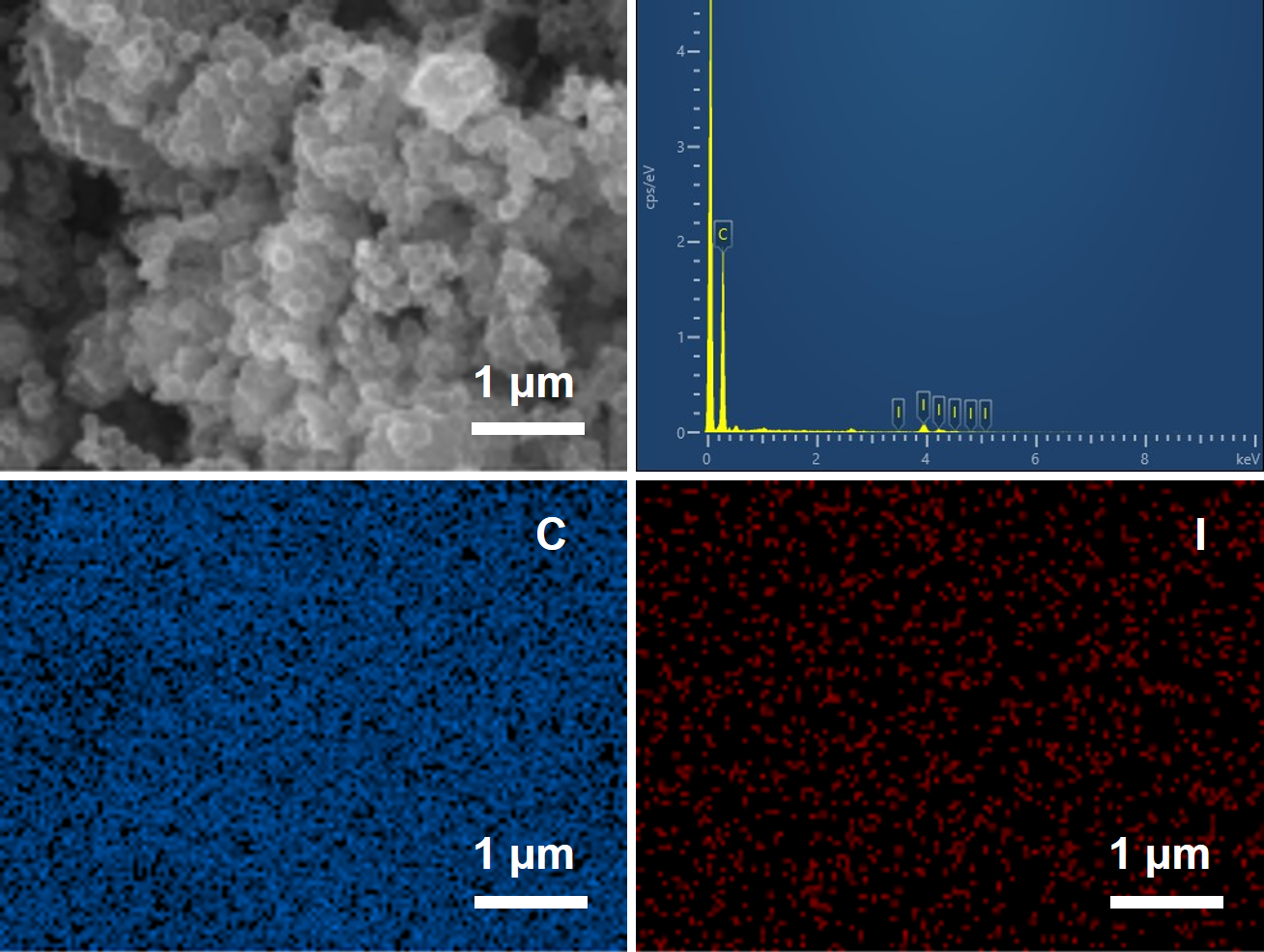


**Fig. S32** EDS image of NC@I_2_


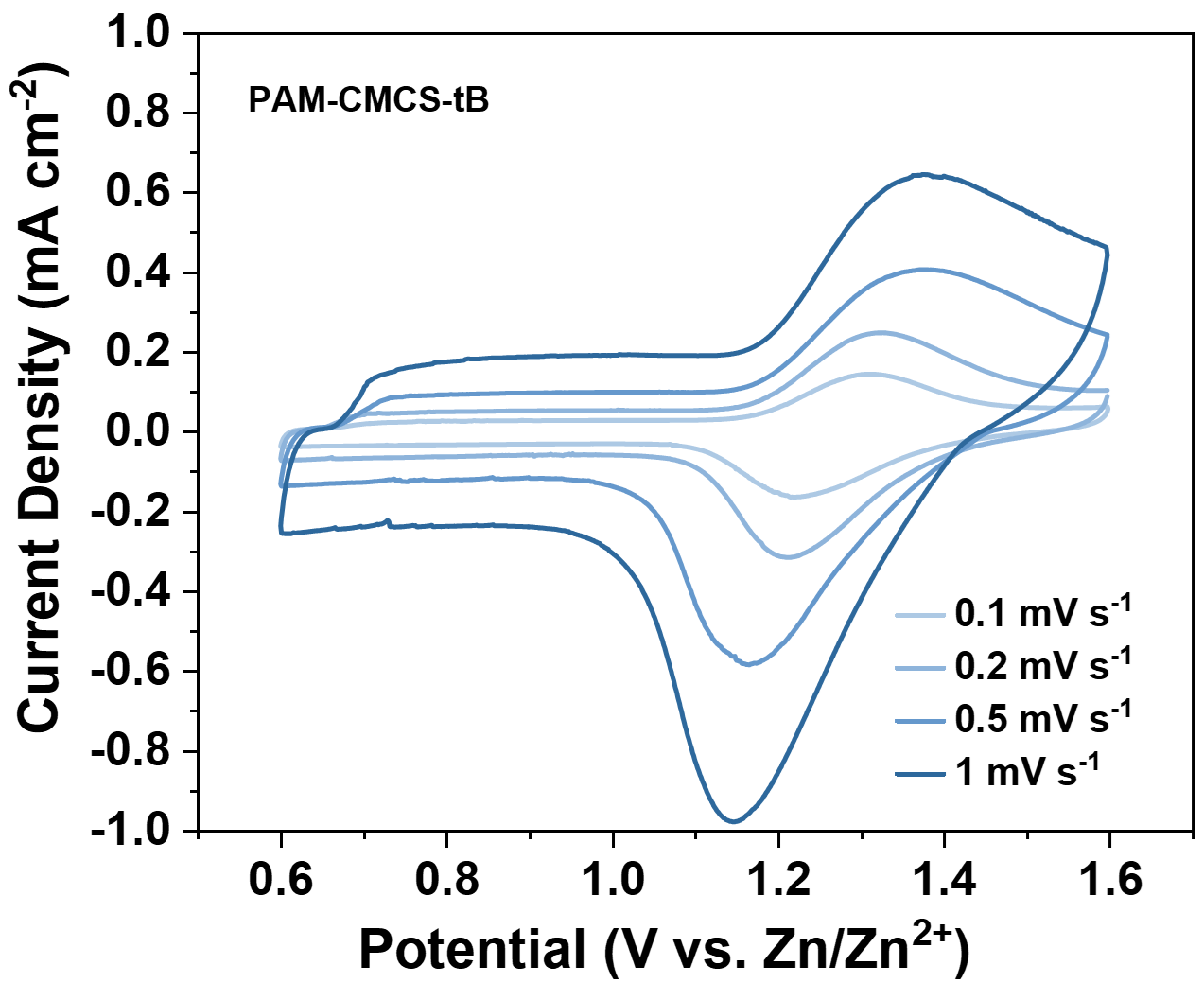


**Fig. S33** CV curves of Zn/PAM-CMCS-tB/I_2_ cells at different scan rates


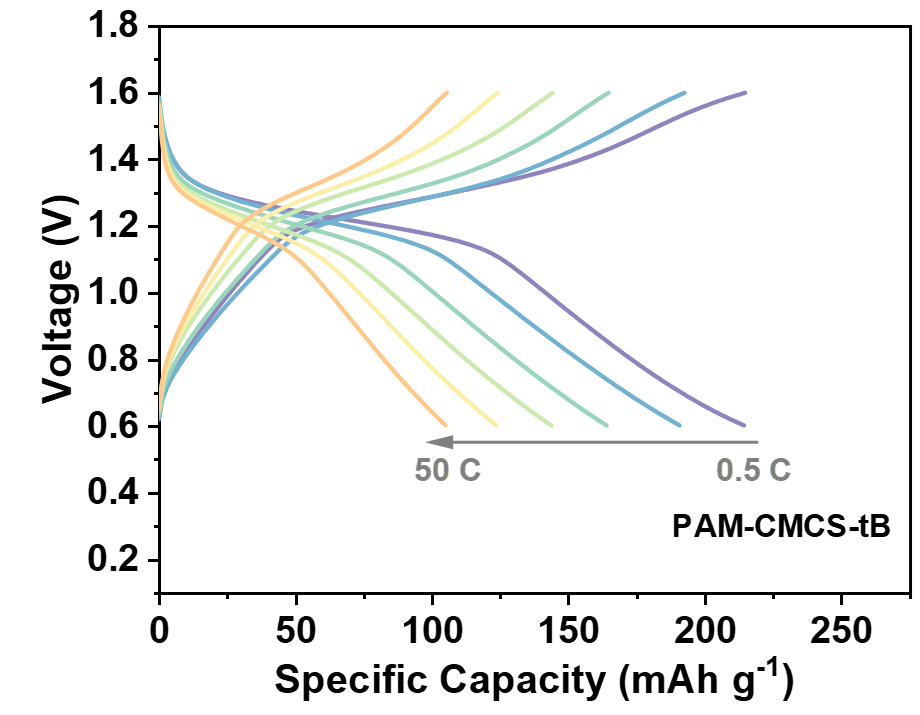


**Fig. S34** Charge-discharge profiles at various current densities


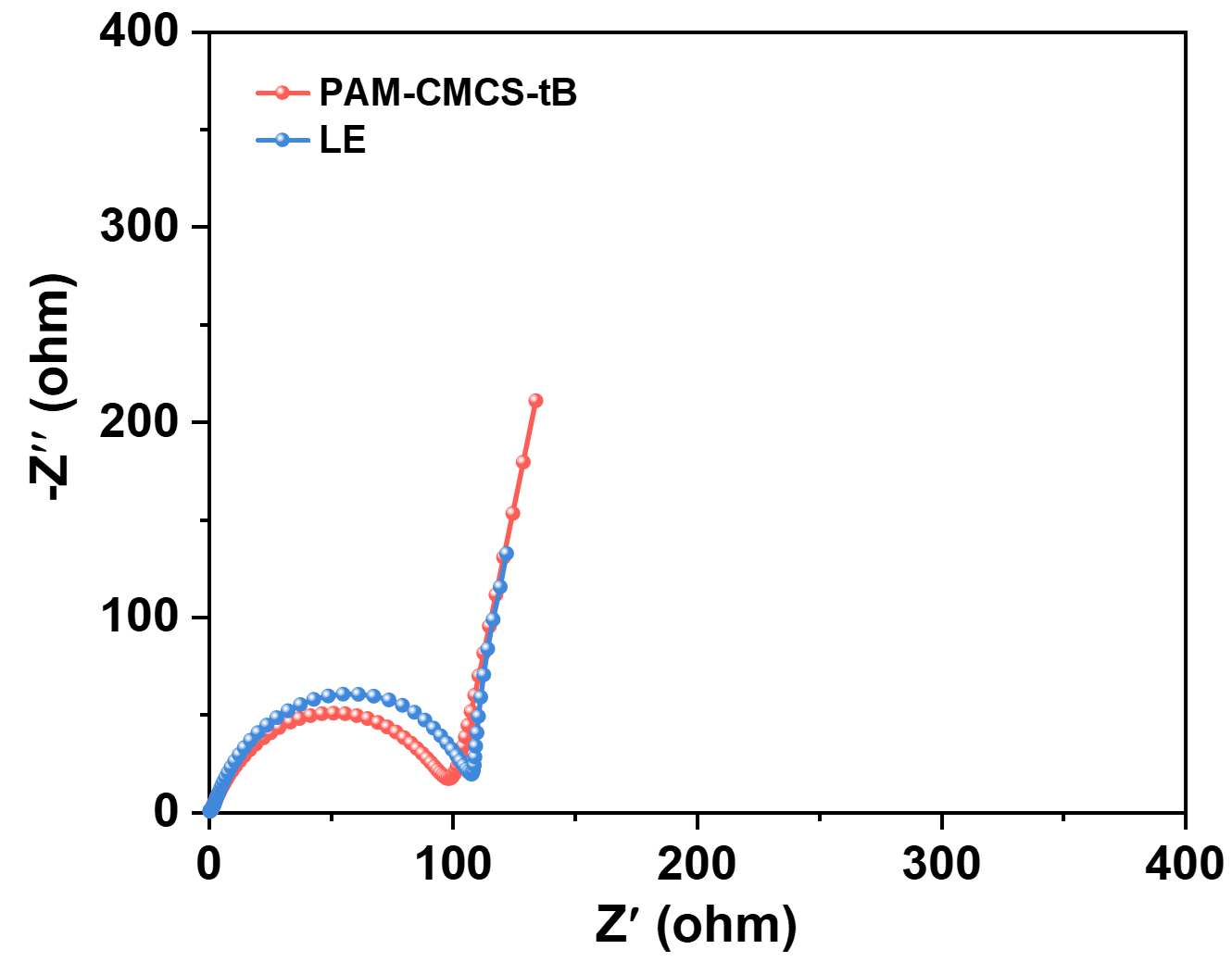


**Fig. S35** Nyquist plots of Zn-I_2_ full cells


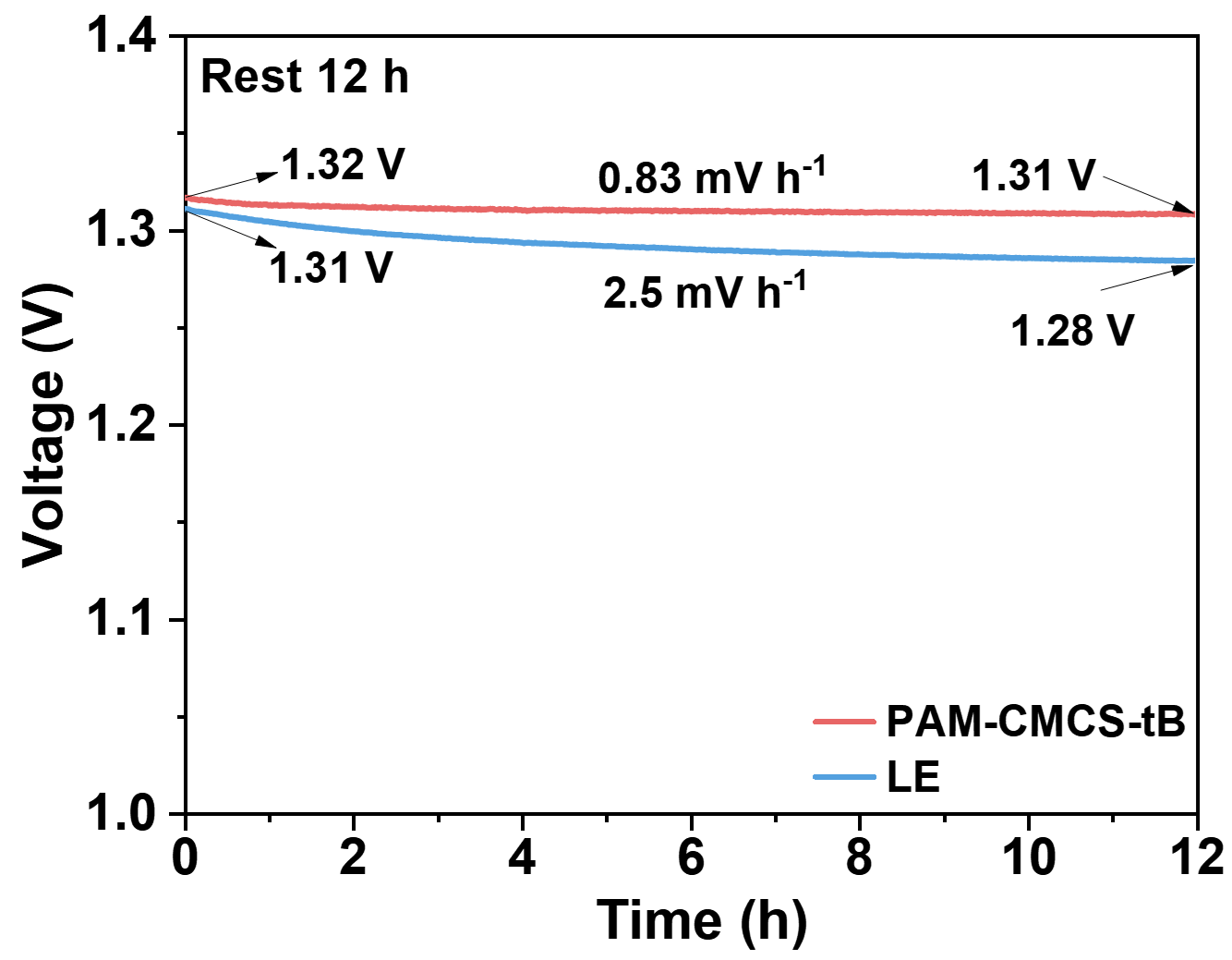


**Fig. S36** Static evaluation of the initial state of charge (SoC)


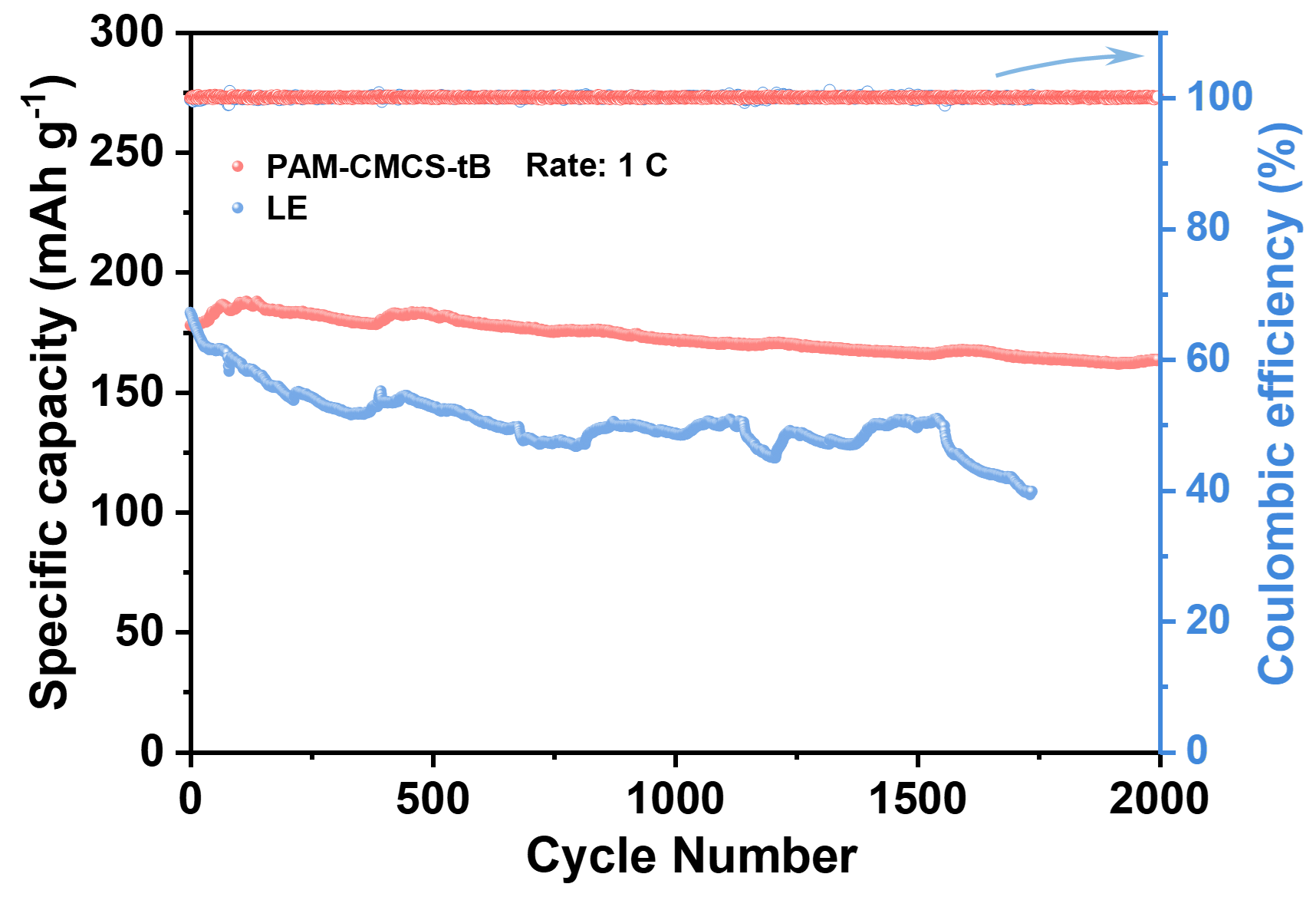


**Fig. S37** Cycling performance at 1 C rate


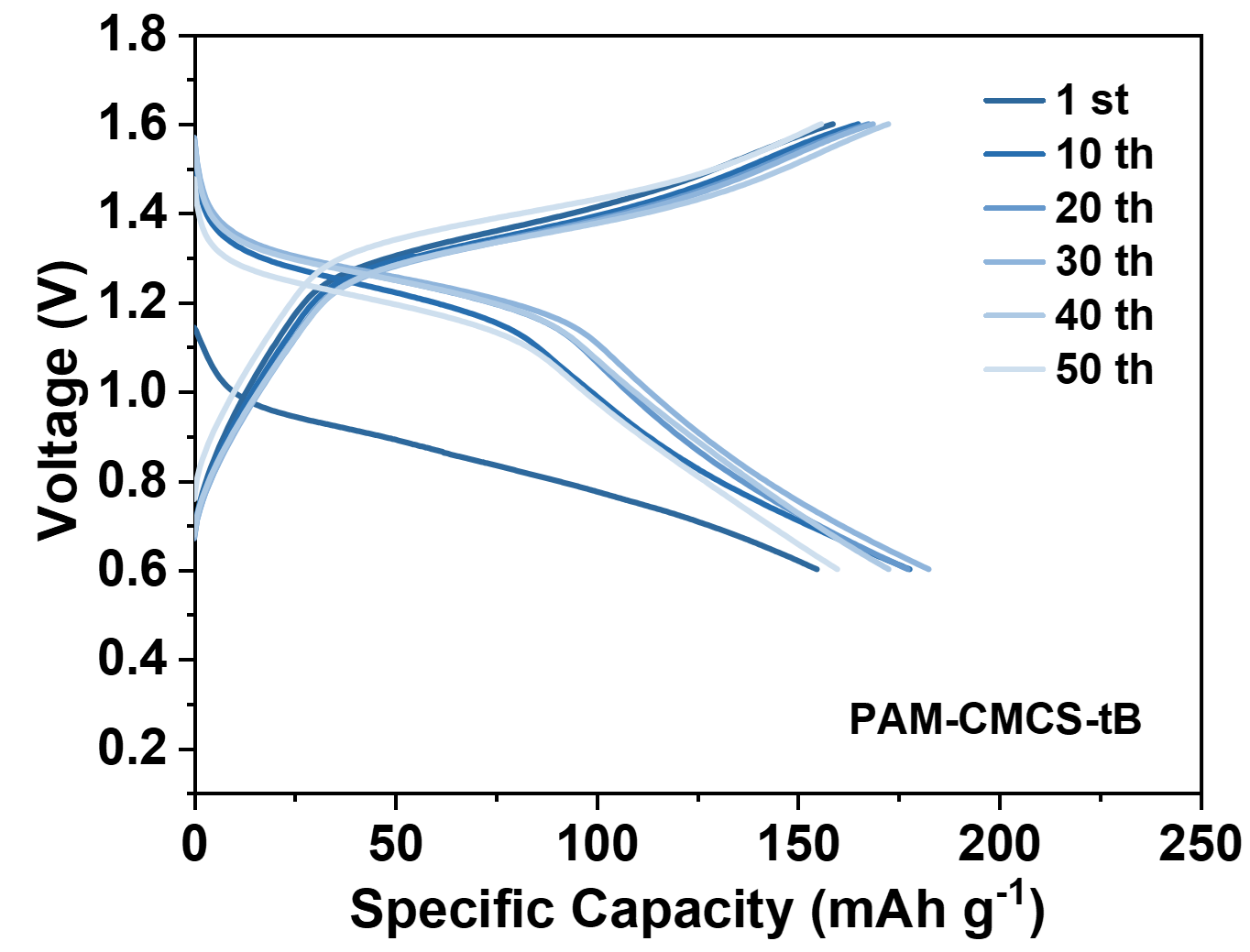


**Fig. S38** GCD curves of Zn/PAM-CMCS-tB/I_2_ cells at 1 C rate for different number of cycles


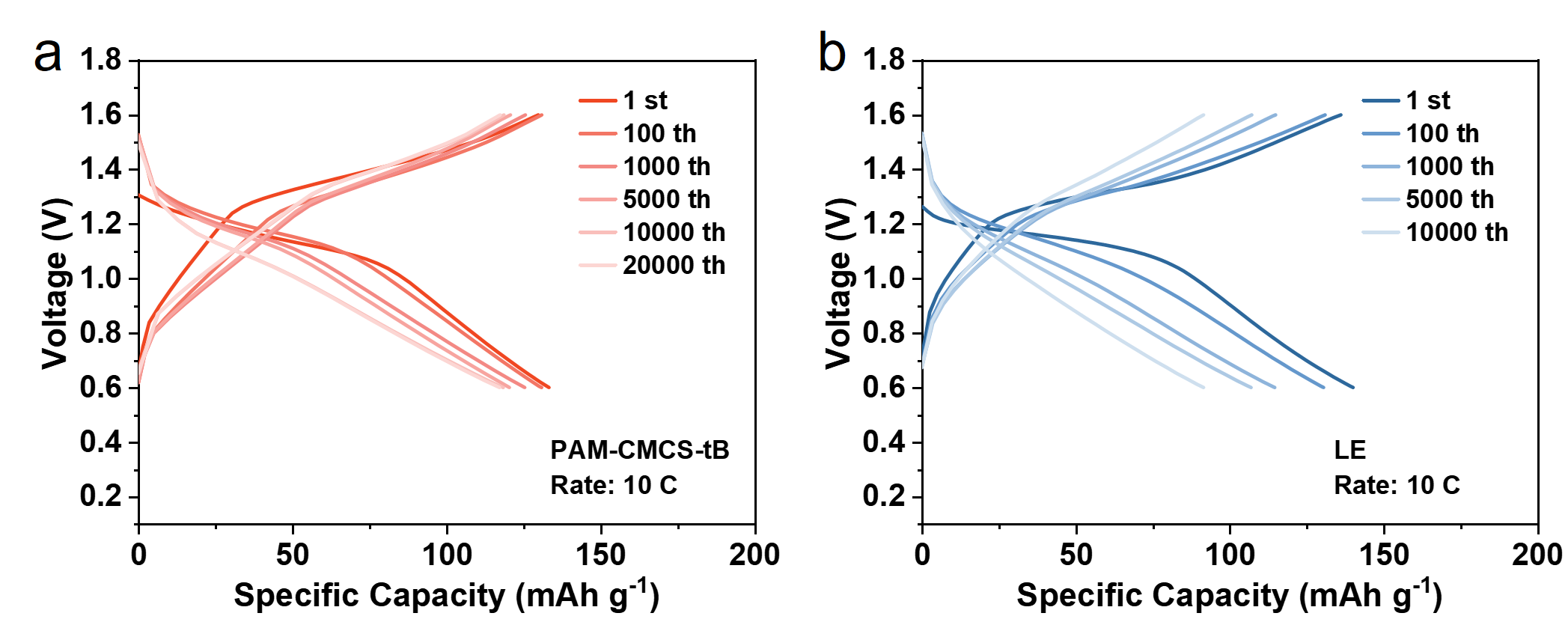


**Fig. S39** GCD curves of **a** PAM-CMCS-tB and **b** LE electrolytes at representative cycles.


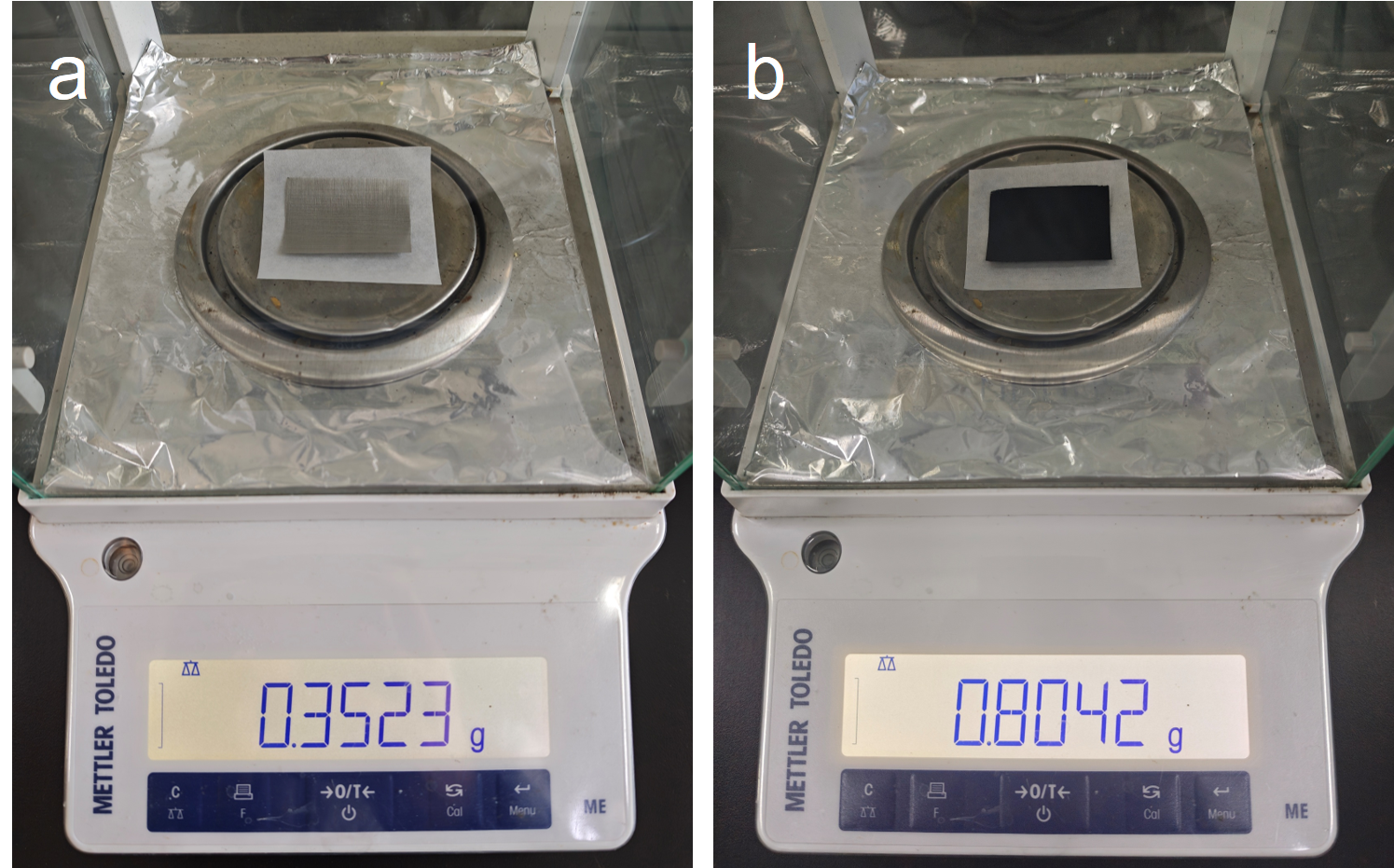


**Fig. S40** Optical photos of NC@I_2_ cathode (cropped to 3.5 × 4.5 cm)


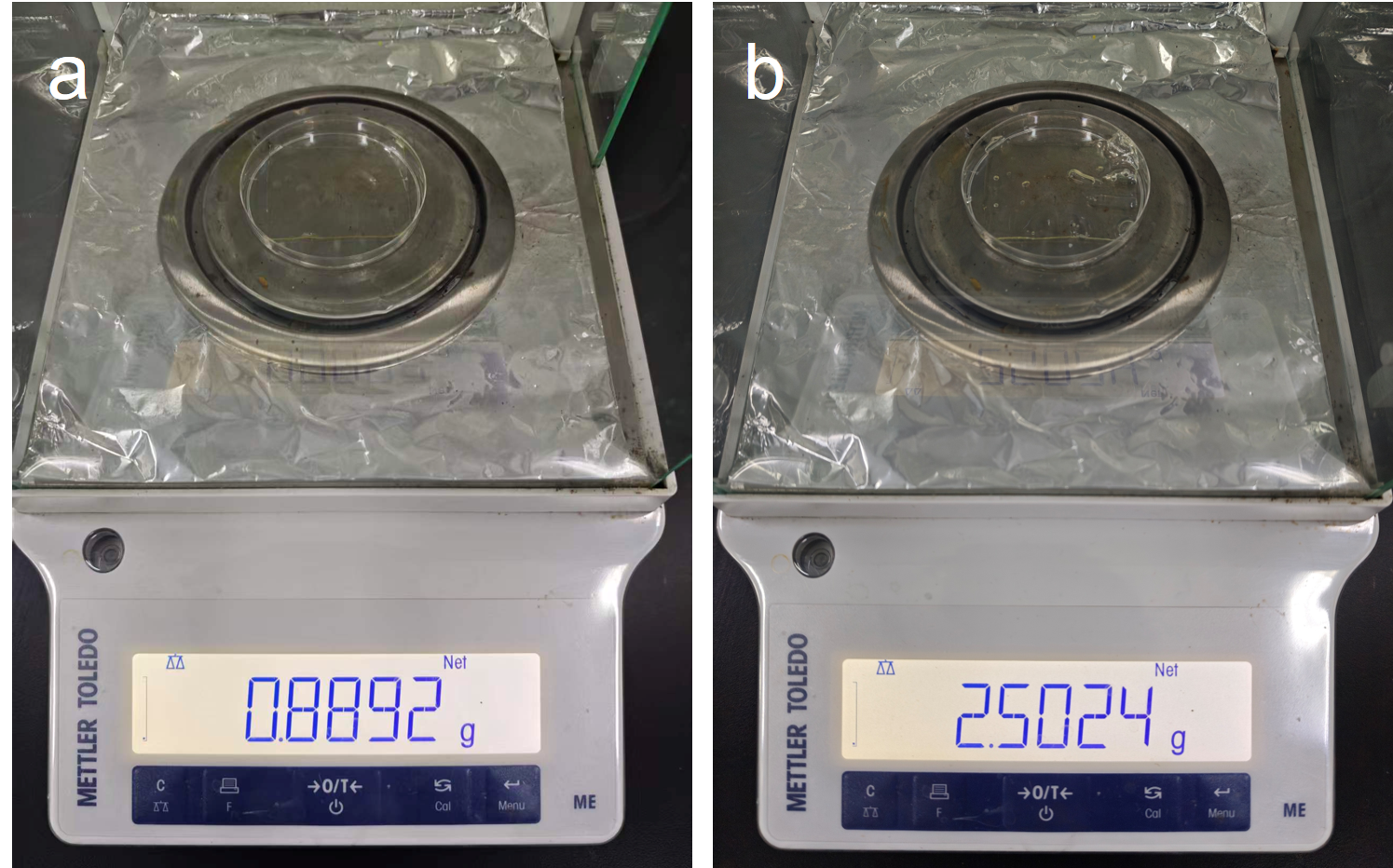


**Fig. S41** Optical photos of gel electrolyte before and after immersion in 2 M ZnSO_4_ (cropped to 4.0 × 5.0 cm)


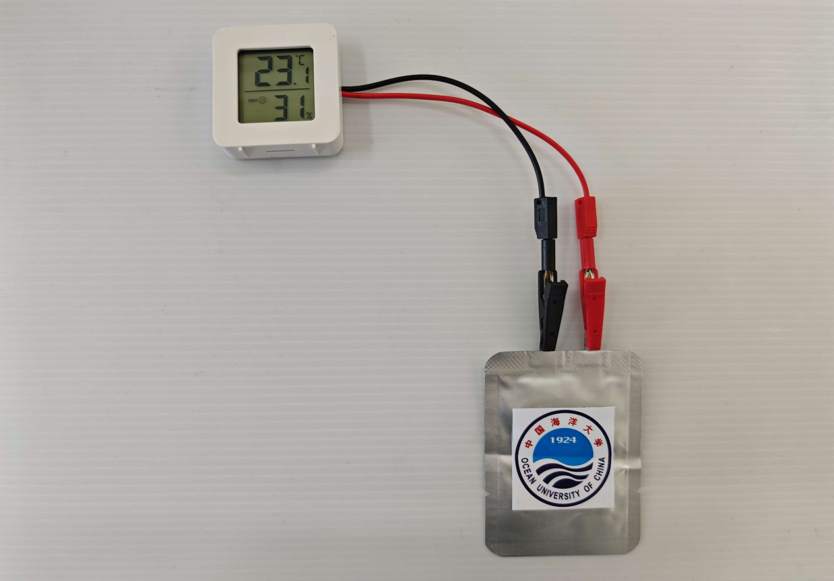


**Fig. S42** Optical image of the monitoring device powered by a PAM-CMCS-tB hydrogel-based pouch cell

**Supplementary Tables**

**Table S1** Mechanical properties and ionic conductivity of the dual-network hydrogel

| Electrolyte | Tensile strength (kPa) | Fracture strain (%) | Ionic conductivity (mS cm^-1^) | Ref |
| --- | --- | --- | --- | --- |
| HACC-co-PAM | ≈150 | 590 | 16.8 | [S1] |
| PNMA/SA | 838 | - | 33.1 | [S2] |
| PAPTMA | 96 | 680 | 28.7 | [S3] |
| G/PAAm/AG | 72.4 | 489.2 | 26.4 | [S4] |
| This work | 30.7 | 518 | 18.1 |  |

**Table S2** A summary of the advanced performance of the recently reported Zn-I_2_ battery

|  | Symmetrical cell | Full cell |  | Refs. |
| --- | --- | --- | --- | --- |
| Electrolyte | Lifespan  (mA cm^-2^;  mAh cm^-2^; h) | Cycle performance  (A g^-1^; mAh g^-1^; cycles; retention rate) | Reaction type (electron transfer) |  |
| Hydrogel Electrolyte (ZnSO_4_) | 1; 1; 2100 | 2; 94.1; 1000; 96.4% | 2 | [S5] |
| Hydrogel Electrolyte (ZnSO_4_) | 0.5; 0.5; 3500 | 20; ≈100; 85000; 90.1% | 2 | [S6] |
| Hydrogel Electrolyte (ZnSO_4_) | 1; 1; 3000 | 2 C; 200; 2000; 82% | 2 | [S7] |
| Hydrogel Electrolyte (ZnSO_4_) | 1; 1; 5000 | 2 C; 205; 2000; 82.9% | 2 | [S8] |
| Hydrogel Electrolyte (ZnSO_4_) | 1; 0.25; 6300 | 2; 185.6; 8000; - | 2 | [S9] |
| Hydrogel Electrolyte (ZnSO_4_) | 5; 5; 1385 | 5 C; 287.8; 3700; - | 4 | [S10] |
| Hydrogel Electrolyte (Zn(ClO_4_)_2_) | 1; 1; 2000 | 2 C; 200; 2000; 100% | 2 | [S11] |
| Hydrogel Electrolyte (Zn(ClO_4_)_2_) | -; -; - | 1; ≈610; 4000; 91% | 4 | [S12] |
| Hydrogel Electrolyte (ZnCl_2_) | 5; 2; 1200 | 1 C; 215.1; 1000; 96.5% | 2 | [S13] |
| Hydrogel Electrolyte (ZnCl_2_) | 0.2; -; 2400 | 0.2; ≈150; 4500; - | 4 | [S14] |
| Liquid Electrolyte (ZnSO_4_) | 2; 2; 1800 | 0.2; 219.3; 1000; 88.7% | 2 | [S15] |
| Liquid Electrolyte (ZnSO_4_) | -; -; - | 20; 138; 63000; 95% | 2 | [S16] |
| Liquid Electrolyte (ZnSO_4_) | 1; 1; 7000 | 1; 158.6; 13000; 75.2% | 2 | [S17] |
| Liquid Electrolyte (ZnSO_4_) | 0.5; 0.5; 2250 | 4; 303.4; 6000; 96% | 4 | [S18] |
| Liquid Electrolyte (ZnSO_4_) | 1; 1; 2100 | 1 C; ≈410; 600; 93.4% | 4 | [S19] |
| Hydrated Eutectic Electrolyte (ZnSO_4_) | 5; 5; ≈600 | 2 C; 404.6; 3000; 76.2% | 4 | [S20] |

**Supplementary References**

1. L. Yang, C. Xu, L. Liu, Y. Li, Z. Chen et al., Network-reinforcing HACC-co-PAM hydrogel electrolytes for suppressed zinc dendrite growth and high-performance zinc-ion batteries. EES Batteries **1**(3), 633-639 (2025). <https://doi.org/10.1039/D5EB00055F>
2. W. Zeng, S. Zhang, J. Lan, Y. Lv, G. Zhu et al., Double network gel electrolyte with high ionic conductivity and mechanical strength for zinc-ion batteries. ACS Nano **18**(38), 26391-26400 (2024). <https://doi.org/10.1021/acsnano.4c09879>
3. D. Lin, Y. Lin, R. Pan, J. Li, A. Zhu et al., Water-restrained hydrogel electrolytes with repulsion-driven cationic express pathways for durable zinc-ion batteries. Nano-Micro Lett. **17**(1), 193 (2025). <https://doi.org/10.1007/s40820-025-01704-5>
4. C. Ji, X. Lin, Y. Hong, J. Liu, A. Liu et al., A robust dual-network hydrogel electrolyte coupled with a porous carbon material for flexible quasi-solid-state zinc ion hybrid supercapacitors. New J. Chem. **48**(19), 8753-8762 (2024). <https://doi.org/10.1039/d4nj00310a>
5. X. Zhang, J. Li, F. Xie, X. Xu, X. Sun et al., Polyzwitterionic gel electrolyte: Dual optimization of polyiodide shuttle suppression and anode stabilization in aqueous Zn‐I_2_ batteries. Adv. Funct. Mater. **35**(39), 2505132 (2025). <https://doi.org/10.1002/adfm.202505132>
6. L. Tang, J. Gao, J. Xu, C. Shao, T. Mu et al., Liquid metal synergistic polyanion self-adaptable gel electrolyte for stabilizing dual electrode/electrolyte interfaces in ultra-durable Zn‐I_2_ batteries. Energy Storage Mater. **84**, 104786 (2026). <https://doi.org/10.1016/j.ensm.2025.104786>
7. J. L. Yang, Z. Yu, J. Wu, J. Li, L. Chen et al., Hetero‐polyionic hydrogels enable dendrites‐free aqueous Zn‐I_2_ batteries with fast kinetics. Adv. Mater. **35**(44), 2306531 (2023). <https://doi.org/10.1002/adma.202306531>
8. J. L. Yang, T. Xiao, T. Xiao, J. Li, Z. Yu et al., Cation‐conduction dominated hydrogels for durable zinc-iodine batteries. Adv. Mater. **36**(21), 2313610 (2024). <https://doi.org/10.1002/adma.202313610>
9. Y. Wang, K. Zhong, J. Zhang, Q. Li, Z. Zhan et al., Concomitant zinc dendrite mitigation and iodide shuttle confinement: A bifunctional zwitterionic hydrogel electrolyte unlocking ultralong‐cycling aqueous zinc‐iodine batteries. Adv. Funct. Mater. e26937 (2025). <https://doi.org/10.1002/adfm.202526937>
10. R. Liu, D. Wang, Z. Gao, D. Yang, Y. Li et al., Low-self-discharge nanoconfined hydrogel electrolyte for stable high-energy-density aqueous zinc-iodine batteries. ACS Nano **20**(6), 5217-5227 (2026). <https://doi.org/10.1021/acsnano.5c20669>
11. Y. Liu, F. Li, J. Hao, H. Li, S. Zhang et al., A polyanionic hydrogel electrolyte with ion selective permeability for building ultra‐stable Zn‐I_2_ batteries with 100 °C wide temperature range. Adv. Funct. Mater. **34**(29), 2400517 (2024). <https://doi.org/10.1002/adfm.202400517>
12. Y. Liu, L. Zhang, L. Liu, Q. Ma, R. Wang et al., All‐climate energy‐dense cascade aqueous Zn‐I_2_ batteries enabled by a polycationic hydrogel electrolyte. Adv. Mater. **37**(46), 2415979 (2025). <https://doi.org/10.1002/adma.202415979>
13. S. J. Zhang, J. Hao, H. Wu, Q. Chen, C. Ye et al., Protein interfacial gelation toward shuttle‐free and dendrite‐free Zn-iodine batteries. Adv. Mater. **36**(35), 2404011 (2024). <https://doi.org/10.1002/adma.202404011>
14. Z. Hu, Z. Han, H. Liu, X. Jiang, K. Bai et al., Mechanically strong and tough ionic liquid gel electrolyte for four-electron zinc-iodine batteries. J. Am. Chem. Soc. **147**(50), 46632-46641 (2025). <https://doi.org/10.1021/jacs.5c18431>
15. W. Liu, H. Ma, L. Zhao, W. Qian, B. Liu et al., Anionically-reinforced nanocellulose separator enables dual suppression of zinc dendrites and polyiodide shuttle for long-cycle Zn‐I_2_ batteries. Nano-Micro Lett. **18**(1), 59 (2025). <https://doi.org/10.1007/s40820-025-01921-y>
16. F. Wang, R. Ma, Z. Chen, T. Yin, Z. Yan et al., Click chemistry‐inspired fixation catalysis for long‐life zinc-iodine batteries. Adv. Mater. **38**(2), e11980 (2025). <https://doi.org/10.1002/adma.202511980>
17. Y. Tan, W. Xu, F. Yang, J. Tao, D. Li et al., Dynamic polyiodide‐trapping and proton‐capturing dual‐network engineering for high‐areal‐capacity, long‐cycling and high‐temperature Zn‐I_2_ batteries. Adv. Mater. **38**(12), e22609 (2026). <https://doi.org/10.1002/adma.202522609>
18. K. Du, H. Qi, C. Zhang, W. Xu, D. Chen et al., Halogenated organic acid additive rendered highly reversible four‐electron transfer zinc‐iodine batteries by strengthened interhalogen interaction and protons supply. Adv. Funct. Mater. e27634 (2025). <https://doi.org/10.1002/adfm.202527634>
19. H. Wu, S. J. Zhang, J. Vongsvivut, Y. Jiang, J. Hao et al., Quasi‐solid cathode additive enables highly reversible four‐electron I^-^/I^0^/I^+^ conversion in aqueous Zn‐I_2_ batteries. Adv. Mater. **38**(3), e11680 (2025). <https://doi.org/10.1002/adma.202511680>
20. L. Liu, L. Zhang, Y. Liu, S. Zhang, R. Wang et al., Enhanced redox kinetics of aqueous I^-^/I_2_/I^+^ conversion chemistry in hydrated eutectic electrolyte over a wide temperature range. Adv. Energy Mater. **15**(38), 2501460 (2025). <https://doi.org/10.1002/aenm.202501460>
